# Imagined futures for livestock gene editing: Public engagement in the Netherlands

**DOI:** 10.1177/09636625221111900

**Published:** 2022-08-01

**Authors:** Senna Middelveld, Phil Macnaghten, Franck Meijboom

**Affiliations:** Utrecht University, The Netherlands; Wageningen University & Research, The Netherlands; Utrecht University, The Netherlands

**Keywords:** anticipatory public engagement, focus groups, gene editing, livestock, narrative, responsible innovation, the Netherlands

## Abstract

Gene editing is an emerging technology with diverse applications in the making, including in livestock. While the technology is commonly represented as offering unbounded possibilities and societal benefit, it remains unclear how to characterise public views and the process through which responses are developed. Rather than simply being about individual attitudes, beliefs or preferences, we explicate an interpretative approach that seeks to understand how people make sense of the technology in the form of shared cultural idioms and stories. Based on five anticipatory focus group discussions with Dutch publics, we found the prevalence of five narratives shaping public talk, namely, technological fix, the market rules, in pursuit of perfection, finding the golden mean and governance through care. We explore the implications of these findings for governance and reflect on the virtues of sophrosyne and phronesis as offering ways to reconfigure the practice and politics of gene editing.

## 1. Introduction

Gene-editing technologies offer novel possibilities to change the molecular structure or function of a gene in targeted ways. Recent innovations, notably the CRISPR–Cas system, have made ‘editing of the genome much more precise, efficient, flexible, and less expensive relative to previous strategies’ (National Academy of Sciences, Engineering and Medicine (NASEM), 2017: 1). These techniques enable researchers to activate or deactivate genes, insert DNA from the same or other species with desirable traits and/or remove parts of DNA that contain undesirable traits (Netherlands Commission on Genetic Modification (COGEM), 2018). Since gene editing is used on living organisms that contain DNA, the technology can be used across species (micro-organisms, plants, animals, humans) and domains of application, including in livestock breeding (Reardon, 2016). Indeed, gene editing has been discussed in natural science and policy debates as ‘a revolution in livestock breeding’ (Ruan et al., 2017: 724), with applications ranging from the breeding of hornless cows, pigs resistant to the PRRS virus, cows that are tolerant to heat and chickens resistant to avian flu, none of which can easily be achieved with conventional breeding techniques (COGEM, 2018; Nuffield Council on Bioethics, 2016).

Even with potential to transform livestock breeding, the technology raises ethical and societal questions concerning our relationship with animals, the social treatment of uncertain knowledge and the limits of human intervention, alongside unresolved questions of efficacy.^1^ For such reasons, national advisory bodies, ethicists, social scientists and natural scientists have called for broad public dialogue on gene-editing technologies (COGEM, 2018; Doudna, 2015; Jasanoff et al., 2015; NASEM, 2017; Nuffield Council on Bioethics, 2016). Early public engagement is an ingredient in the new scientific governance (Irwin, 2006) that gives citizens voice in making governance institutions responsive and accountable (Cornwall, 2008), increasing the legitimacy and quality of decision-making (Holdren et al., 2011), and part of anticipatory governance (Guston, 2014). Yet, few attempts at public engagement on gene editing have been instigated, including on livestock applications. In this article, we respond to this challenge through empirical research that seeks to answer the following question: How (if at all) does the Dutch public imagine a future for gene editing in livestock, and if so, under what conditions? Our contribution is twofold: to deepen our understanding of public responses to the prospect of gene editing in livestock, and to explore how such understanding could contribute to the practice and politics of gene editing. We build on the concepts of anticipatory public engagement, responsible innovation and narrative to explicate our goals.

Anticipatory public engagement uses methodologies aimed at engaging publics in conversations about science and technology at an early stage, when the problem definition, concerns and positions of publics are being configured, rather than settled (Sykes and Macnaghten, 2013; Wilsdon and Willis, 2004). It investigates public responses by deliberating on factors that shape responses, how concerns emerge, how experiences come to matter, and to derive an endogenous account of the societal and ethical stakes (Macnaghten, 2021; Wynne, 2006). For agricultural biotechnologies, the dominant policy narrative has framed regulation and governance in terms of measurable risks, as if public issues of concern can be reduced to specific harms to health and the environment (Gaskell et al., 2004; Jasanoff, 2000; Macnaghten and Carro-Ripalda, 2015; Wynne, 2001). Yet, as acknowledged in policy reports on biotechnology and gene editing (European Group on Ethics in Science and New Technologies (EGE), 2021; NASEM, 2017; Nuffield Council on Bioethics, 2016), this frame is poorly equipped to think through wider societal and moral questions. As the European Group on Ethics in Science and New Technologies (EGE, 2021) stated in a recent opinion on gene editing, ‘ethics should serve to tackle broad governance questions about how technologies can serve our common goals and values, and not be limited to providing a “last step” of “ethics clearing” of a technology’ (p. 5).

Frameworks of responsible innovation are aimed at extending debates on responsibility in science and innovation to their collective and external impacts, covering foreseen and unforeseen effects, alongside an assessment of goals, purposes and ends (Owen et al., 2012). Four dimensions of responsible innovation – anticipation (A), inclusion (I), reflexivity (R) and responsiveness (R): the AIRR framework – provide a scaffold for raising, discussing and responding to questions of societal concern, characteristic of a reflexive vision of innovation (Stilgoe et al., 2013). In this article, the anticipation and inclusion dimensions are central, to open up the debate before gene editing in livestock becomes locked in dominant framings and commitments.

To understand how public responses to gene editing in livestock are constructed, we used an analytical approach that attends to the interpretative and narrative dimensions of public talk (Macnaghten et al., 2019). If we think of public responses not as the aggregate of individual attitudes and preferences but as the struggle towards a vocabulary with which to attribute meaning, the concept of a narrative resource can situate ‘novel developments in ways that render them culturally, morally and affectively sensible’ (Kearnes et al., 2014: 246). This framework leads to a focus on the stories and narratives that are mobilised in public talk in the act of making sense of an emerging technology. Our argument is that narrative resources are mobilised in culture, providing a resource for publics to collectively attribute meaning to the issues and dilemmas posed by a new technology or innovation.

The structure of the article is as follows. In section 2, we elaborate our anticipatory focus group methodology. In section 3, we discuss our narrative approach and present results in the form of five narratives that structured public deliberation. In section 4, we discuss the implications of this analysis for the practice and politics of gene editing in livestock.

## 2. Methodology

We deployed an anticipatory focus group methodology aimed at anticipating the kinds of worlds that gene editing in livestock would bring into being, designed to allow the public to negotiate the meanings of issues endogenously. Following the method presented by Macnaghten (2017, 2021), five design features were followed to ensure quality, relevance and legitimacy: *context, framing, moderation, sampling and group design* and *analysis and interpretation*.

*Context* is a feature of the method given that the public is not familiar with gene editing. It thus becomes necessary to explore the proximal relationships out of which public responses are likely to emerge, also important for framing discussions in ways that do not privilege expert perspectives (Macnaghten, 2017).^2^ We used relations to pets and other animals to start the conversation. If few participants had pets, we used the consumption of animal products as an entry point. In both cases, relationships with animals were used to find common ground and to facilitate group formation. All groups articulated caring relationships with pets and/or other animals. This is unsurprising, since care for animals is widely embedded in Dutch society (Council on Animal Affairs (RDA), 2019), and this remained important throughout the rest of the conversation.^3^

*Framing* is important since technologies are inevitably framed in different ways for different purposes. In the design, we selected an inclusive range of attributed frames that reflected how the issue is discussed by industry, government, non-government organisations (NGOs) and scientists. The frames were about the technology and what it could mean in the present and future (Felt et al., 2014). We prepared a topic guide to guide the discussions (see Supplemental Material A). The moderator presented the frames on pre-designed concept boards (see Supplemental Material B1)^4^ that consisted of pictures and text to stimulate the conversation. While the specific materials inevitably shaped subsequent conversations, in-depth critical engagement with these frames helped groups develop their own problem definitions and meanings. The group discussions were a co-production process between participants and the moderator, where the moderator provided input, but where participants discussed the provided input in an endogenous manner.^5^ Often participants did voice similar concerns, but on occasions they agreed to disagree and were engaged in co-constructing perspectives. None of the groups reached unanimous views about gene editing in livestock, which was not the goal of the discussion.

The *moderator* played a role in facilitating the discussion, keeping the participants on topic, listening to their stories, making explicit similarities/differences, articulating shared definitions of issues, summarising topics and moving onto the next only once the discussion was exhausted. The moderator was important in managing group dynamics. Some groups contained participants who voiced their opinions in a dominant manner. The moderator then intervened giving the floor to more silent participants.

*Sampling and group design* is the fourth feature. Here, we selected groups that represented prototypical segments of the Dutch public from different demographic backgrounds, age ranges, localities and interests. We also introduced topic-specific variables to facilitate distinctive perspectives (see Table 1). These included a group of public sector professionals (to discuss global perspectives), a group of countryside dwellers (to discuss farming and rural life perspectives), a group of health and well-being enthusiasts (to discuss health and bodily perspectives), a group of young parents (to discuss local community perspectives) and a group of private sector professionals (to discuss corporate perspectives). A professional recruiting company recruited five groups with eight participants per group. A financial incentive was offered for participation in the study. The participants had no a priori stakes in the debate (we did not include gene-editing experts) and were unknown to each other. Recruitment was ‘topic blind’. Participants were told they would take part in a discussion on societal issues. Participants’ consent was obtained in advance to record the sessions, and discussions lasted for approximately 2:30 hours. The first group was moderated by the second author in English; the other groups by the first author in Dutch.

**Table 1.** Selection criteria used to recruit focus group participants.

Group	Name	Age	Gender	Place	Topic-specific variable
1	Public sector professionals	45–65	4F/4M	Amsterdam	Global citizens
2	Rural group	30–60	4F/4M	Amersfoort	The countryside
3	Food and outdoor enthusiasts	25–45	8F	Amersfoort	Health and well-being
4	Young parents	25–45	5F/3M	Amsterdam	Local community
5	Private sector professionals	30–55	8M	Amsterdam	Technology

Following the focus groups, recordings were transcribed and translated into English. Transcripts were coded and analysed generating codes and themes. All team members worked on the transcripts, meeting periodically to review the themes, exchanging ideas about the analysis. From the thematic categories, we explicated a set of narratives that participants drew upon in structuring responses. In the analysis and interpretation, we were attentive to how talk is shaped by the narratives, and when the narratives are shaped by the talk. Since both are co-produced, we cannot approach narratives as static or fixed but as performative and in the making. For data interpretation, we became well-acquainted with the data through ‘an iterative process of reading, thematic coding and reflection, . . . to identify a series of key themes and sub-themes’ (Williams et al., 2017: 93).

## 3. Results and analysis

For our analysis, we built on the idea of narrative as explicated by Agnes Heller (2005) in her study of European narratives on freedom:

[master narratives] are the stories to which we always return, they are the final, or ultimate foundations of a type of imagination. Yet, as the guides of imagination, they also rule, control and are vested with power. Direct and indirect references to master narratives provide strengths and power to new stories or new images. (p. 257)

For Heller (2005), narratives are performative and embodied in everyday practice, ‘emotionally felt, without footnotes, without explanation or interpretation’ (p. 257). A narrative approach is an analytical tool for understanding how public responses to an unfamiliar topic emerge, ‘a space to excavate the terrain of culture, with the aim of understanding how arguments surrounding novel technologies form, persist, endure and possibly fade’ (Kearnes et al., 2014: 247; for other examples, see Davies et al., 2009; Motion and Doolin, 2007). Public responses showed participants struggling towards a vocabulary to make sense of gene editing in livestock and its implications. Our focus on narratives proved analytically helpful to codify and make explicit the implicit, to engage with moral, cultural and technological meanings and questions.

The narratives drawn upon in the discussions were not new. As reflected in previous research, we found narrative resources to originate ‘in ancient classics and philosophy and have found ongoing and enduring expression in the form of fables, morality tales, literature, and more recently in films, science fiction, and video games’ (Macnaghten, 2010: 33). Examples of ancient narratives are the ‘Trojan horse’, or ‘Be careful what you wish for’, that draw in complex ways on ancient stories of desire, evil and the sacred (Dupuy, 2010). How ancient narratives are adapted and translated to modern times becomes an empirical matter, including their (new and old) roles and functions. We find that next to ancient narratives, participants draw upon modern narratives such as those of exploitation (‘The rich get richer’) or alienation (‘Kept in the dark’), where the purposes and social impacts of a technology are scrutinised (Dupuy, 2010).^6^

We found that people drew upon a complex interplay of narratives to make sense of gene editing in livestock. Four broad concerns structured public responses: the purpose of the technology, the fair distribution of benefits, uncertainty and risks and governance (also see Macnaghten and Chilvers, 2014). These concerns came forward after coding the transcripts and are part of the stories told by participants about gene editing. We identified five narratives that people drew upon in articulating concerns and which were entangled in different ways. Our task was to identify both the concern that was raised and the narrative story in which the concern was situated. This enabled us to move from a thematic analysis of concerns to an analysis of underpinning narratives and their entanglement. We further elaborate on the narratives in the following text.

### Technological fix

The technological fix narrative is a modern story whereby attempts to respond to social problems by technical means (often created by previous technological interventions) give rise to new problems (often requiring new technological interventions). In the discussions, participants expressed doubt if the application of gene editing in livestock was the right solution to the right problem. Overarching challenges in agriculture included the overconsumption of livestock products and a growing world population. This problem formulation was seen as underpinning an array of global challenges including those of climate change and biodiversity loss. Instead of contributing a solution to these problems, gene editing was described as failing to engage with underlying causes, as in focus group discussion 1 (FGD1) as follows:

Mod:  ‘Scientists have developed this CRISPR–Cas system. They say that gene editing is now more precise, efficient and flexible compared to previous strategies . . .’

Lucy: ‘I see like we are living in a dome; it looks that we are not living in a real world anymore . . . It’s as if we have to use this to survive, we, humans and animals . . . And I would rather not, I’d rather have another way to solve the problems. To heal the world so to speak . . .’

Brian: ‘[W]hat problem are we solving and what are the causes of this problem? . . . [I]t’s still a plaster on a self-inflicted wound. It’s a plaster on something we’ve created ourselves’.

While gene editing in livestock is framed institutionally as offering benefits, participants viewed the technology, even when benignly intended, as more likely to exacerbate persistent and long-term societal and environmental problems, as expressed in FGD3:

Holly: ‘Solving one problem creates another. If the sheep can stay out in the wet season, maybe other problems will arise’.

Ivy: ‘If you are really thinking in the far future, when you are manipulating humans and animals, at some point we would reach ages of 150 or 200 years. And will we still fit on this globe? You may find drought resistant crops, which will feed people, better food also, so people will live longer but I think at one point we will be reaching the capacity of this planet’.

While applications for animal welfare and disease resistance purposes were legitimate goals for the technology, it was less clear whether these goals would materialise. In FGD4, this concern was articulated in a discussion on the dehorning of cows:

Tom: ‘Animal welfare should be the starting point, and not dehorning [of cows] so that they can stand closer to one another. They should give the animals more space so that they don’t need to be dehorned. But it has to do with space, and therefore money . . .’

Jamie: ‘I do not agree [with this application of gene editing], because you should not be dehorning cows. You have to leave them in their own [nature]. . . . so that they don’t grow horns anymore for the sake of productivity. And God has created the cow with horns, there must be a reason for it’.

A similar argument was used for applications aimed at disease resistance which were viewed as an incentive to keep livestock in confined spaces, leading to higher livestock numbers, less space per animal, higher efficiency, and cheaper production. The logic of this narrative is where it may become inevitable that all livestock are gene edited for a range of purposes (welfare, disease resistance, increased yields), and where the technology is used to maintain society’s status quo within the current system, drawing us from the (radical) reconfigurations required in reconfiguring society and livestock relations. The technofix narrative resonates in societal debates about the limits of technological approaches to global challenges: for example, the promise of genetically modified (GM) plants to ‘feed the world’ (Macnaghten and Carro-Ripalda, 2015), or geoengineering as a solution to climate change (Macnaghten and Szerszynski, 2013).

### The market rules

The market rules narrative is predicated on the value of equality, and the potential and likelihood of technology to increase inequality locally and globally. Using this narrative to mobilise critique, participants argued that even though gene editing in livestock may be promoted to be inclusive and generative of societal benefit, the logic of the market will lead only the rich and privileged to benefit, whereas the poor or excluded become marginalised. The use of this narrative was prominent in all the discussions, reinforcing scepticism and unease with the technology, as expressed by participants in FGD1 below, at times causing doubt on the value of public engagement exercises such as this one:

Alex: ‘Is it going to be better only for the rich? Or is it going to be better for all of us in this world? I think . . . it’s going to be for the Western world. And African people or in South America, who are poor, will not have access to these solutions. That can make me angry sometimes . . .’

All: ‘It’s all about the money really . . .’

Martin: ‘We need some time; it’s going all too quickly. And the aspect of money always keeps coming back. . . . And public opinion [can be] a trap because, you are letting us express ourselves here, but we don’t know what our influence is going to be’.

A related argument was made by participants in FGD5 on questions of access:

Tim: ‘Maybe, this way we could turn the Sahara into the breadbasket of the world’.

Peter: ‘What I fear is that the big breakthrough [i.e. crops that can grow] in the Sahara, is that it could end up as a patent for one huge corporation. Most of the time they will go for the big money . . . I think if big money will be involved, you’re going to create bigger gaps between the haves and the have nots’.

Similarly, participants in FGD3 were not convinced that access to the technology would trickle down to wider society:

Sally: ‘If this is going to be available, it will only be for the Kardashians of this world. I don’t think it’s going to be available for the average person’. . ..

Nina: ‘I find this the presentation of the ideal world. Tomatoes that need less water are a high-tech thing. A normal farmer cannot use that. So, my fear is that it will be reserved only for a small group of companies, or farmers. And they will make a lot of money with it’.

In short, an increase in inequality is an undesirable future for our participants, perhaps not surprising given the historical importance attributed to the value of equality in Dutch society. Such concerns are part of a wider history of concern and protest to structural inequalities arising from agricultural biotechnologies, ranging from large-scale protests against multinationals in Europe to GM crops and foods (Levidow, 2000), to mobilisations in countries as diverse as Brazil, India, Mexico and South Africa (Macnaghten and Carro-Ripalda, 2015; Scoones, 2008).

### In pursuit of perfection

Not foreseeing the consequences of one’s actions is the concern associated with the pursuit of perfection, a motivation (not always explicit) seen as endemic in the development of gene-editing technology through its ambition at producing ‘better’ animals (and ‘better’ humans). Through our mastery of nature, the argument runs, we desire to live according to ideals of perfection, where imperfections become attributed to choice rather than chance, and life no longer represented as a gift (Sandel, 2004). Across all groups, this narrative was adopted to stress how the pursuit of perfection in animals through gene editing would inevitably lead to a striving for perfection in humans, and vice versa, both with negative consequences. Given this association, the use of this narrative inflected a more human focus. Fostering a harsher and more self-absorbed society was viewed as a consequence that arises from the pursuit of perfection, as discussed by participants in FGD3:

Janet: ‘I think that society is going to be tougher, because our expectations are going to become higher. Everything is going to be perfect: your meat is perfect, your vegetables are perfect, your body is perfect. In the future, everything imperfect will be tougher to handle . . . Even today, . . . I find society quite tough already; I think it’s going to become even harsher’.

Mod: ‘According to you, are there any things that should be forbidden?’

Winny: ‘I think that you need to look at the individual desires that cannot be fulfilled. You should also let magic do its thing. If we are going to determine everything in life, it will be less pleasant, I think’. . . .

Rosa: ‘It is so nice today that we humans are all different, isn’t it? And maybe in 500 years from now we are all perfect people, don’t you find that a bit chilling? I’m afraid that we will be losing each individual’s uniqueness’.

By contrast, diversity was a value to be cherished. Temporally, participants in all the groups connected the pursuit of perfection narrative with the violent history of eugenics. Interestingly, Dutch memories of the legacy of World War II and of Nazi eugenic practices remain salient in the Netherlands today. As Taussig (2004) comments in her analysis of Dutch responses to a transgenic bull known as Stier Herman (Herman the bull) two decades back, this reference remains pertinent since ‘[m]ost Dutch people consider Nazi eugenics the complete antithesis of the highly valued Dutch social ideal of tolerance’ (p. 308). Across the focus groups, participants drew on eugenics as a cautionary tale of the dangers of using genetics that would lead us down a ‘slippery slope’ (Meloni, 2016), as in this extract from FGD1:

Maya: ‘If this were to become legal worldwide, there will be even more abuse. It’s going to explode; everyone with particular genes and with money can buy the perfect child. And this is the onset to move towards immortality. . . . Everything in this is scary, because everyone wants to be perfect, and, it’s sounding so romantic, but we got to accept, we are just humans’.

For this group, we need to be careful what we wish for, because such individual and hedonistic desires could reinforce a host of societal and spatial problems. The alternative is to accept that ‘we are just humans’, with inevitable imperfections and mortality.

### Finding the golden mean

While the three narratives above characterise dangers associated with institutional and corporate logics and practices, the next two articulate the conditions under which the technology could in principle be developed responsibly. The finding the golden mean narrative originates from a tension between the desire to master nature and humility for nature, or what in ancient Greek philosophy has been depicted as the vice of hubris and the virtue of sophrosyne. Deploying this line of thought, Sandel (2004) argues that the danger of genetic engineering lies in its: ‘Promethean aspiration to remake nature, including human nature, to serve our purposes and satisfy our desires’ (p. 5), and how this disrupts an appreciation for ‘the giftedness of life’. Hubris represents ‘a misguided and inflated conception of oneself and one’s place in the world’ (Cairns, 1996: 8), to consider yourself equal or superior to a god and to attract divine interference, especially when motivated by an excessive pursuit of honour and status.

The belief that gene editing was complicit with a hubristic attitude was shared in the discussions. The hubris embedded in the technology and proposed applications lay with a sensed superiority of science to nature and its tacit ordering. The narrative relies on the ancient proposition that if we display hubris, we transgress our moral boundaries (Schroten, 1992), leading God or nature to strike back (Macnaghten et al., 2019), as discussed in FGD5:

Mod: ‘Is it ethical at all to use the technology?’

Peter: ‘No, because you are tampering with nature. And you do not know what the long-term effects are. You may knock out a particular gene for peanuts, but you don’t know what the effects of that are going to be in 10 years from now’. . . .

Clint: ‘It is still a bit controversial because you’re going to play God in a way. . . So, the idea is noble, but it’s also leading to many questions’.

Central in this quote are the unknown long-term effects of gene editing arising from its potential to intervene in nature in new and radical ways. This generates unresolved questions: Off-target effects? Health effects on consuming edited livestock products? Long-term effects? Central to the narrative is the likelihood of unanticipated effects, and a warning not to pursue the technology without due care and attention. Participants in FGD2 deployed this narrative to justify caution:

John: ‘Up to now we only had the natural DNA follow its course, and now we are intervening in the DNA?’

Evy: ‘Yes, that is what I’m saying’.

John: ‘And what’s going to happen if you cut the wrong part?’

Evy: ‘And if you eat it, what’s going to happen then? What is this DNA going to do with you, inside you?’

Rich: ‘I am also afraid of that’.

Jesse: ‘Me too, I agree, this is a breach into natural processes’.

Evy: ‘This is not nature anymore’.

The other dimension is the virtue of sophrosyne. Being moderate (sophron) requires we avoid excess, accept our limits, possess self-knowledge and avoid overextension, including in our technological capabilities (Rademaker, 2005). To practice sophrosyne, we practice the golden mean through ‘a cautious and gradual process of trial and error’ (Stanisevski, 2015: 11). We found participants articulated concerns by drawing on the virtue of sophrosyne: to be modest and humble, to take time and care in making decisions, to acknowledge imperfect knowledge and accept our (technological) limitations. This is expressed in FGD2 in relation to the complex functioning of nature in diseases and infertility:

Evy: ‘[I]t’s not for nothing that sometimes genes are not working properly, and diseases do appear. This is a conscious defect, built into nature to prevent overpopulation. . . . If you are not fertile, it is harsh what I am saying now, but that’s what nature has foreseen’.

Mark: ‘I think you are right; I think we should let nature do its thing’.

This discussion is premised on the belief that nature has a way of balancing life and that the temperant act may be one of accepting things as they are. In FGD3, tampering with nature was associated with the excessive use of the technology for the purpose of increases in meat production. In FGD4, participants rejected the application of gene editing to breed hornless cows since hornless cows can be bred with existing breeding techniques. This demonstrates a practical interpretation of finding the golden mean, in this case the belief that if alternatives to gene editing are available (i.e. through natural breeding), then these are preferred. Finding the golden mean is also deeply embedded in Dutch culture and politics in the form of the ‘polder model’, a slow and consensus-based form of decision-making in which diverse stakeholders make pragmatic decisions despite differences (Van der Meer and Hemerijck, 2016).

### Governance through care

Concerns over the representation of the technology as promising far-reaching societal benefits were tempered with apprehension that the technology in practice would lead to unforeseen and negative consequences. For our participants, and for these reasons, we need to exercise care in developing responsible governance. Phronesis (practical wisdom) is a virtue required in decision-making processes: the ability to assess a situation and choose the best way forward. Phronesis is learned through experience, through deliberating on values and normative demands when thinking through practical problems (Mejlgaard et al., 2019). Applying the concept is helpful in asking the following questions: ‘Where are we going? Is this desirable? What should be done? Who gains and who loses? And through which mechanisms?’ (Flyvbjerg, 2001: 60). We find phronesis a useful concept to elaborate on discussions on governance. All groups were in favour of a deliberative model of governance of independent experts with authority to decide about gene editing in livestock. FGD5 discussed this as follows:

Clint: ‘A normal person cannot assess this, let alone regulate this. Therefore, I think it should be a scientist, that is aware of the consequences, to weigh up the pros and cons, and it’s going to take quite a while before they make any decisions. . . . By scientists, we also mean independent experts. And they have to study the thing in a project that is being set up by the State, not just any private initiative. Also, philosophers need to be in the committee’.

This quote reflects a preference for a governance model that is deliberative, independent and composed of experts with knowledge to foresee consequences. Such a model emphasises phronesis in decision-making, an important criterion being whether the technology is desirable for the world at large. This will take time, and sophrosyne. All groups agreed that industry – from pharmaceutical companies to breeding companies – should not be involved in the governance process. FGD5 discussed it as follows:

James: ‘The pharmaceutical industry should not be part of it, or else you are going to be dealing with the pharma mafia, because this is pure gold! And the meat industry will also be pushing these bulls with a lot of muscle mass. That’s what they want’.

In FGD3, it is stressed that we need time to make good decisions given the formidable social and ethical stakes:

Janet: ‘I would suggest thinking about this for a very long time, by a huge group of people in a multidisciplinary team, so that things turn out to be okay. Today it looks small, but one wrong decision can lead to a great many numbers of disasters’.

Aligned to the call for taking decisions carefully, all groups expressed confidence in the Netherlands as a nation and culture well-equipped to govern the technology responsibly. Perhaps parochially, the further from the Netherlands, the greater the perceived danger of irresponsible governance, presenting formidable challenges for developing authoritative governance at the global level. For this reason, both the Netherlands and the European Union are seen to play a formative role, as discussed in FGD3 in the following text:

Alice: ‘I suppose that here in the Netherlands it is going to be well governed, but I have less confidence in other countries’.

Tammy: ‘There should be worldwide agreements about this. Some kind of code of conduct should be created internationally . . . with an institute that inspects and manages it all’.

In other groups, participants expressed concerns about unethical use, such as the gene-edited babies born in China, or military uses of the technology, for example, by nations such as Russia. Public deliberation points to the need for global agreement about the purposes for which the technology is allowed to be used, as well as evaluation of likely consequences. Such arrangements require global cooperation, and a framework for negotiating differing values and perspectives. When the technology is developed slowly, and when decisions about purposes and applications are carefully made, it becomes plausible to arrive at possible agreements. What is rather specific to the Netherlands is the high trust placed in Dutch government to make the right decisions,^7^ which was not the case for British citizens who felt ‘Kept in the dark’ by their government and industry when nanotechnology applications were silently introduced to the market in face creams (Macnaghten, 2010).

## 4. Discussion

So far, we have sought to deepen our understanding of public responses to the prospect of gene editing in livestock by exploring the stories that people draw upon in developing responses. Our proposed typology does not cover all public concerns, and there are certainly other narratives which people draw to develop views and attitudes. Nevertheless, the five narratives emerged consistently across the discussions and are a robust means of understanding the formation of responses in guided social interaction.

How do our results compare with previous research? There are clear parallels with analogous examples of ‘upstream’ public engagement that include GM crops and foods (Grove-White et al., 1997), animal biotechnology (Macnaghten, 2004), nanotechnology (Macnaghten, 2010), synthetic biology (Pauwels, 2013; TNS-BMRB, 2009) and geoengineering (Corner et al., 2013; Macnaghten and Szerszynski, 2013) with closest parallels to the cultural narratives examined in the DEEPEN project on nanotechnologies (Macnaghten et al., 2019). Across each domain of techno-visionary science and innovation participants questioned the motivations driving the innovation, their (foreseen and unforeseen) capacity to cause ill and harm, the prevalence of market logics and their likelihood to accelerate inequality. This finding is itself surprising as it points to a widespread rejection of a master narrative of techno-science in guaranteeing social progress across an array of technological domains. Other surprises pertain to specificities of Dutch culture and society: the link between the pursuit of perfection narrative with eugenics, the high trust placed in the Dutch government when compared with other jurisdictions, the importance of finding the golden mean through the Dutch tradition of the ‘polder model’, as well as the general eloquence and thoughtfulness of the conversations.

What do the salience and use of these narratives from our Dutch publics imply for the politics and policy of gene editing in livestock? In the Netherlands, and Europe more generally, the application of gene editing in livestock is far from commercialisation. In the European Union, all applications involving genetic modification (that following a 2018 ruling from the European Court of Justice includes gene edited organisms, see CJEU, 2018) are subject to a rigorous authorisation process in which they are assessed for potential risks to human health and the environment. In addition, the Netherlands has supplementary regulations governing animal biotechnology, including a ‘no, unless’ policy in which applications are subject to a compulsory ethical review, with emphasis on how any intervention impacts on animal integrity, and only accepted if no realistic alternatives are available (COGEM, 2018). Such an ultra-cautious approach would no doubt meet the approval of our Dutch focus group participants, none of whom appeared enthusiastic about the prospects of gene edited livestock any time soon. Nevertheless, the focus group discussions provided some indications of the conditions under which people might become open to the prospect of gene edited livestock, such as conditions of equality or if the technology is part of a system change.

From our analysis, we used the term phronesis as the practical wisdom necessary for responsible governance. This may seem contrary to claims that the notion of practical wisdom that focuses on finding the middle ground is intrinsically at odds with the disruptive character of innovation (Blok, 2019). However, the results from the discussions show that expectations towards government are not restricted to procedural or legal solutions, but often start in expectations of the moral attitude of a government, for example, to protect citizens from harm or strive for justice. These are not static principles but responsibilities that require practical wisdom to make operational decisions. Therefore, dealing with gene editing in livestock requires of governance actors to practice moderation (sophron) in evaluating and thinking through the impacts of emerging technologies, opening scientific (episteme) and technological (techné) knowledge to public norms and values that move society towards responsible gene editing. Practical wisdom is essential to deal with various dimensions of governance, especially when innovations come with disruptive consequences. In current policy debates, phronesis is rarely a central tenet, which is a sign of an underlying issue. Policy tends to be informed by the natural sciences and framed around measurable risks and benefits. Yet, such a frame is strikingly incongruent with the tenor of conversation experienced in our focus group discussions. On the contrary, the public narratives we encountered transcend questions of technical risk or of managing the technology. They involve a broader societal and moral questioning of technology, including questions about equity, access, the limits of market logics, the desirability of goals, and whether gene editing might inadvertently exacerbate a host of persistent societal problems.

A classical response to the tension between technocratic governmental frames and societal responses is to make modifications only within the existing frame, resulting in discussions of the same kind. This can be recognised in ongoing governance discussions on whether gene editing is equivalent to genetic modification from a scientific perspective (Leone, 2019; Macnaghten and Habets, 2020). The problem is that no matter how detailed or nuanced the analysis it has two shortcomings. First, it starts from a technical approach rather than from practical wisdom aiming at balancing all relevant dimensions (cf. Schwartz, 2011). Second, it overlooks the underlying cause that underpins the response in the first place. The widespread unease unearthed by our focus group participants may be part of a wider recognition of the lack of limits emblematically signalled by gene editing technologies and their radical capacities to transform what defines us as humans and animals.

These foundational questions are rarely on the policy agenda, or in the way governance is practised. Thus, the pertinence of public narratives implies for the governance of emerging technologies the need to take them seriously and early in the development of a technology and in policymaking, to reduce the likelihood of controversy and to help ensure the technology is developed with and for society (Macnaghten, 2020; Wilsdon and Willis, 2004). How to take public concerns seriously in governance remains an open question in need of research and experimentation. The public did provide suggestions: a group of independent experts should decide, and they need time and care to make good decisions. Companies should not be represented in the governance process (to avoid the pitfalls of the market rules), and care for the planet should be a driving priority. Such processes require finding the golden mean between our desire to master nature (hubris) and acceptance of our limitations (sophrosyne). Humility, modesty and moderation are principles to guide the process (also see Jasanoff, 2003). Enshrining virtues like sophrosyne and phronesis in governance processes is likely to involve both institutional redesign (e.g. through embedding frameworks of responsible innovation) alongside work on cultivating a culture of responsibility through activities that include moral education, ethical training and multidisciplinary collaboration (Grinbaum and Groves, 2013; Reijers, 2020; Stilgoe et al., 2013). We argue that striving for the mean is particularly important when extreme innovations are at stake. Their extremity can have large and irreversible consequences for life on earth, and for these reasons we need to exercise care (and moderation) in decision-making practices, and to be open to alternative solutions to our societal challenges alongside the development and use of new technology.

## Supplemental Material

sj-pdf-1-pus-10.1177_09636625221111900 – Supplemental material for Imagined futures for livestock gene editing: Public engagement in the Netherlands Click here for additional data file. Supplemental material, sj-pdf-1-pus-10.1177_09636625221111900 for Imagined futures for livestock gene editing: Public engagement in the Netherlands by Senna Middelveld, Phil Macnaghten and Franck Meijboom in Public Understanding of Science

## Notes

1. In this article, we explore the ethical and societal questions associated with livestock gene editing. We do not examine debates concerning the efficacy of the technique including the possibility that not all the targeted genes from a genome are edited or deleted, leading to unintended effects. For an examination of these debates, see Nuffield Council on Bioethics (2021).

2. We are grateful to one of the reviewers of this article for bringing this point to our attention.

3. This makes it questionable to claim that care for animals only came forward due to how we started the conversation.

4. We used six concept boards: (1) Four scenarios of livestock farming; (2) What is gene-editing? (3) Human applications; (4) Agricultural applications; (5) Visions of gene-editing in livestock; and (6) Gene editing and governance.

5. We are grateful to one of the reviewers of this article for articulating the importance of this ‘co-production’ conceptual register.

6. Our use of narrative bears a family resemblance to the concept of the sociotechnical imaginary (Jasanoff, 2015; Jasanoff and Kim, 2009) in that both attempt to analyse practices and discourse at the level of culture rather than the individual. While both concepts pertain to entities that are collectively held across groups, a narrative analysis is suited to the analysis of public talk whereas the sociotechnical imaginary concept is suited to compare contrasting institutional discourses (for an analysis of the sociotechnical imaginaries of scientists and breeding companies on gene editing in livestock in the Netherlands, see Middelveld and Macnaghten, 2021).

7. This may be different now, because the focus groups took place before the outbreak of COVID-19.
