# Supplementary material for: Imagined futures for livestock gene editing: Public engagement in the Netherlands
Source: Public Underst Sci. 2022 Aug 1;32(2):143–58. doi: 10.1177/09636625221111900 (PMC9902986; doi:10.1177/09636625221111900)

Title: Imagined futures for livestock gene-editing: Public engagement in the Netherlands

Authors: Senna Middelveld[1], Phil Macnaghten[2] and Franck Meijboom[3]

[1] Division of Farm Animal Health, Department of Population Health Sciences, Faculty of Veterinary Medicine, Utrecht University, the Netherlands

[2] Knowledge, Technology and Innovation Group, Wageningen University and Research, Gelderland, the Netherlands

[3] Division of Animals in Science and Society, Department of Population Health Sciences, Faculty of Veterinary Medicine, Utrecht University, the Netherlands

Corresponding author: Phil Macnaghten
Address: Hollandseweg 1 (Room 4018), 6706KN Wageningen, The Netherlands.
Tel: +31317484018
E-mail address: philip.macnaghten@wur.nl

Supplemental material A: Topic guide

1. *Welcome*

(5 minutes)

2. *Animals in Everyday life*

- What animals do you have (or have had) a particular relationship
    - Perhaps as pets (Or as wildlife (or in another capacity) and tell me one thing you like, and one thing you are not so keen about having them
    - How has having animals affected everyday life?

    What does this involve?

    What is good/ not so good about it?

    What is it that builds a good relationship? (Time?)
- Think of a memorable animal you have known (Probe in what way)

(15 minutes)

3. Animals in society

- What are the key changes in how we, as a society, are using/ treating animals these days? (LIST)

    (Add to List, Vegetarianism, Changing practices in Zoos, re-introduction of beavers, Conservation of wildlife, intensive production of animals for food)

    (RANK in terms of significance)
- Are there trends here?

    What is contributing to these trends?

    Are these conflicting? In what way?
- So eating meat has been mentioned (by a few?)
    - How do you feel about eating meat? And has this changed?
- Is this different from other ways in which you think about animals (e.g. as pets?)?
    - And how does this make you feel?

(20 minutes)

4. *Visions of the Future of Livestock Farming*

- Here are four different visions of the future of livestock farming (on Board 1)
    a. Sustainable intensification: smart farming: precision agriculture, internet of things, big data, monitoring of individual animals, high welfare, incl. gene editing etc.
    b. Intensive model: neoliberal model, high input-output, mechanisation, larger farms etc.
    c. Smallholder model: high-tech, organic, agro-ecology, tightly knit communities, (new) traditions
    d. No livestock model: plant-based alternatives, cultured meat, high tech
- What do you think of each of them?
    - How plausible/ realistic are each of them likely to be?
    - Which do you desire? Prefer?

- What is the role of technology in each model? And what do you think of this?
- How do you think each of these models thinks about animals?

(20 minutes)

5. *What is Gene Editing*

- Introduce the term and the CRISPR-Cas9 system: gene editing and explore meanings

If you looked up a definition in an encyclopaedia it might say something like this (on Board 2)

a. Board on what is gene editing, what it is, how it works

- Have people heard about such developments? Where? What have they heard?
    - Have they discussed them? With whom? Why?
    - Do you have any questions you'd want to ask about these things?

(15 minutes)

6. *Agricultural and Human Applications of Gene Editing*

- Introduce Board 3 on agricultural applications of gene editing:
    - Does anything surprise you? Concern you? Excite you?
    - Which are plausible? And why? What is driving these developments?
    - How do you feel about different bits of the board?
- Introduce Board 4 on human applications of gene editing:
    - Does anything surprise you? Concern you? Excite you?
    - Which are plausible? And why? What is driving these developments?
    - How do you feel about different bits of the board?
- Comparing Boards 3 and 4:
    - How do you feel about differences between the board? (explore differences in animal, plant and human applications)
    - How do the animal/ livestock applications in particular make you feel?

(20 minutes)

7. *Visions of Gene Editing in Livestock*

Here is how three different kinds of actors are thinking about gene editing as applied to livestock (show and display each on Board 5)

a. Positive vision (breeding industry and some academic scientists): enabler of sustainable intensification, better welfare (hornless cows), disease resistant animals, environmental benefits—using arguments of scientists and breeding companies
b. Negative vision (environmental NGOs): a new wave of research on genetically engineered animals is leading us in the opposite direction to sustainable and ecological farming system — by designing animals to better fit within industrial systems rather than addressing the underlying health, animal welfare and environmental problems associated with these systems: Are they necessary, ethical and welfare concerns, unforeseen effects using arguments from NGOs and civil society organisations (including FOE report)

c. Case-by-case vision (bits of government): all display dilemmas; depends on purposes; uses of gene editing in animals will depend on how it is used and for what purposes; that we need a public debate etc.
d. Non-ideal vision (some academics):

- How plausible/ realistic are each of them likely to be?
- Explore differences across different domain areas.
- Do you have any questions you'd want to ask about these things?
- Does anything surprise you? Concern you?
- Which (if any) do you feel an affinity with? Why? Why not?
- Which do you desire? Prefer?

(20 minutes)

8. Gene Editing in Animals and Governance

So, what do you think about governance?
Should it be governed? How? What criteria?
Active debate on how to regulate and govern gene editing in animals, which we will now explore

a. Board on different national approaches to regulation (US on one side, EU on the other), debates in Europe on whether a gene edited organism falls within the GMO directive, appeals to societal debate

Explore responses and reactions
(15 minutes)

9. Future visions and what is at stake

- Can you start to imagine ways these innovations and ideas might change our lives? What kinds of societies might they lead to?
- How do you feel about these?
- What would it mean to live in a world where some of these ideas have become real? How might society adapt?
- Who or what do you think might drive these changes?
- Do you have any especial concerns about these possibilities?
- Is there anything you'd be especially excited about?
- Is there anything you think should not be allowed to happen?
- Is there anything you think might be particularly publically controversial? Why?

(15 minutes)

10. Conclusion

- Explanation of project and feedback.
- How do each of you respond to the question: 'What, if any, are the conditions under which gene editing in livestock is socially acceptable?'
- Final messages.

(5 minutes)

Four Scenarios of Livestock Farming

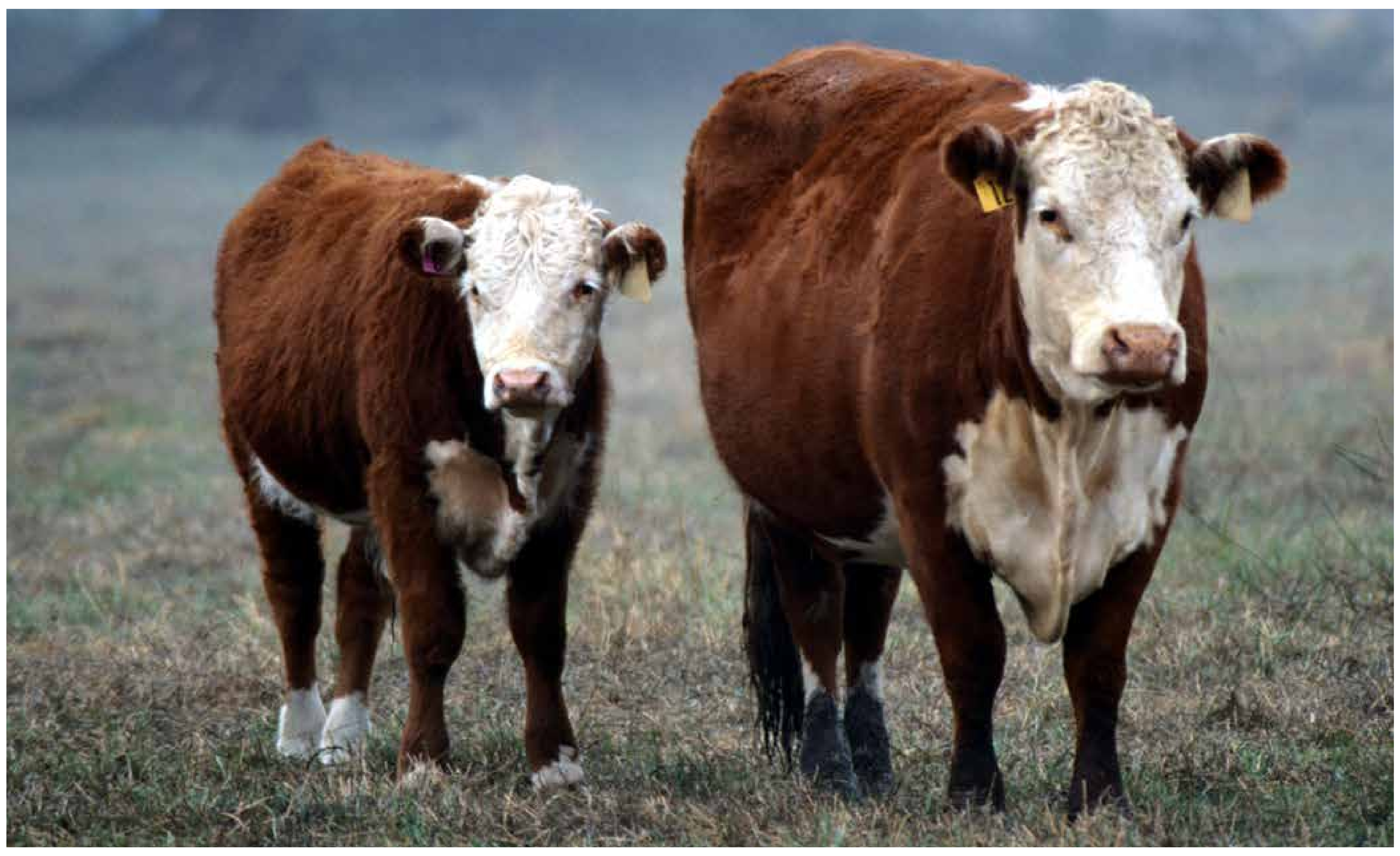

1. Organic Farming

"[A]groecological models of production, including diversified organic and well-managed pasture-based systems, provide a host of benefits."

Friends of the Earth, 2019

2. Industrial Farming

"Given the stakes, the world must deliver on agriculture's full potential. (...) Agriculture is and must continue to be innovation-driven."

World Economic Forum, 2019

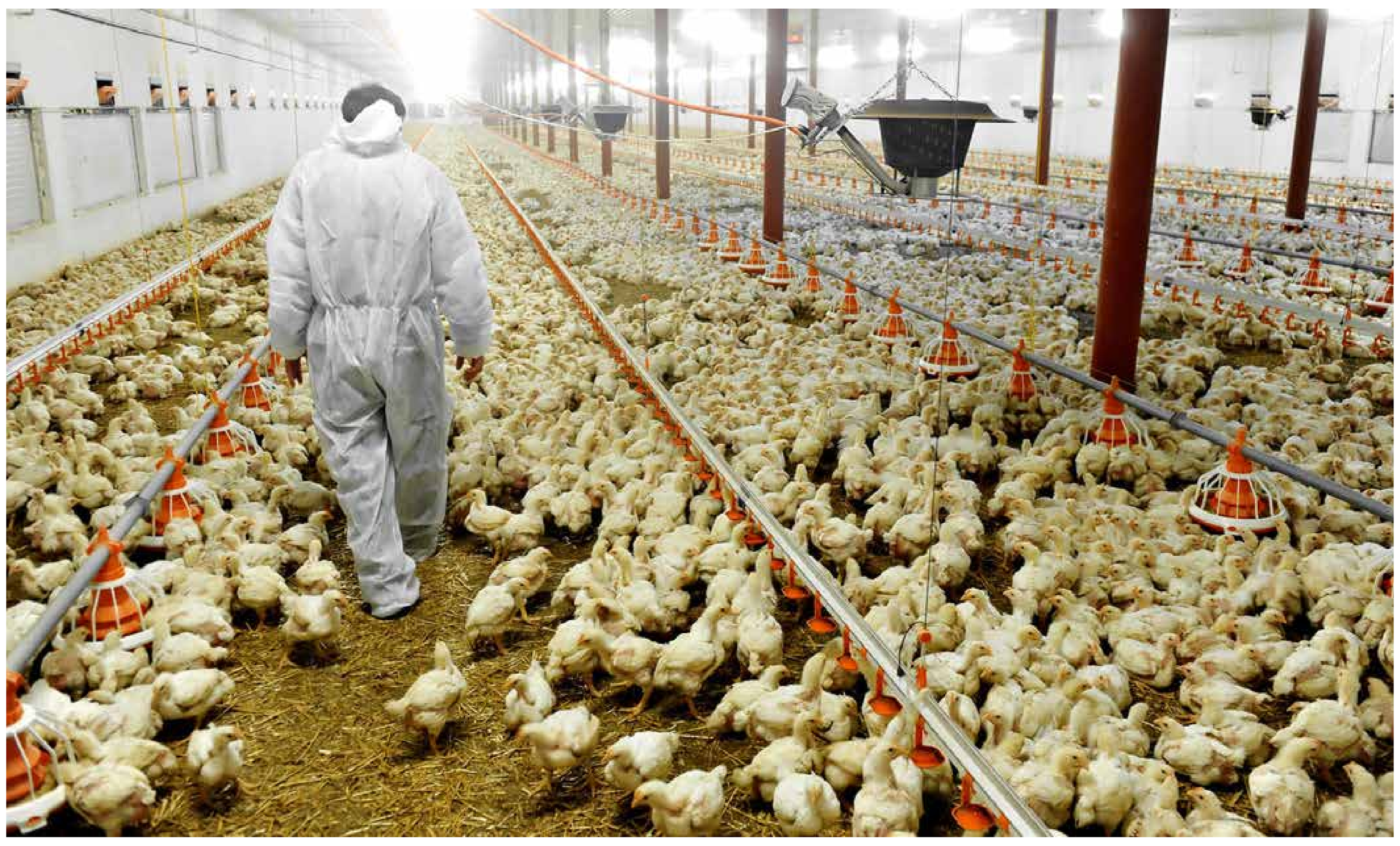

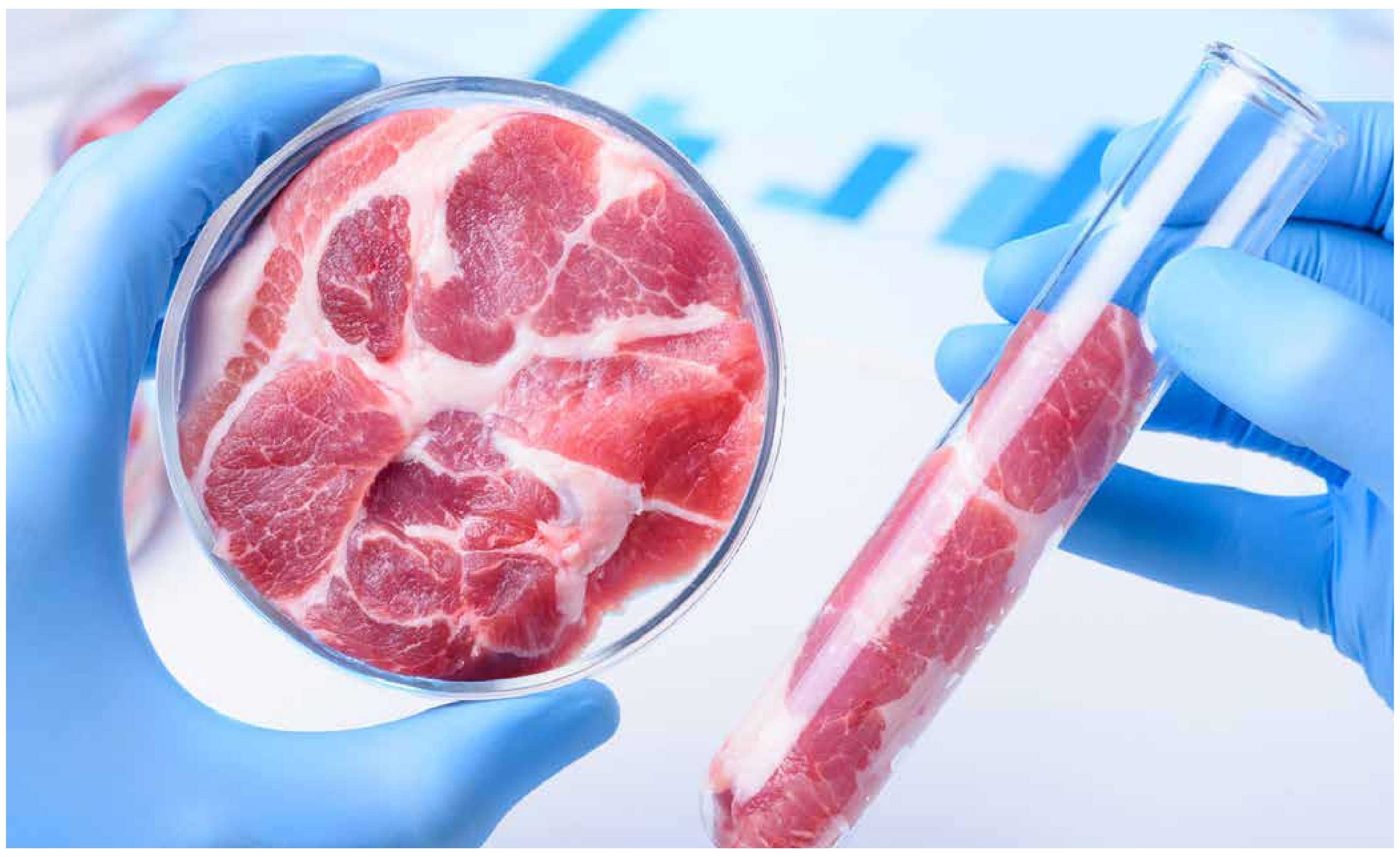

3. No Livestock Farming

"Rising global demand for meat will result in increased environmental pollution, energy consumption, and animal suffering. Cultured meat, produced in an animal-cell cultivation process, is a technically feasible alternative."

Van der Weele & Tramper, 2014

4. Precision Farming

"Precision agriculture (PA) (...) will assess the needs and conditions of individual animals in larger herds and optimise feeding on a per-animal basis. (...) The methods of PA rely mainly upon a combination of new sensor technologies, satellite navigation and positioning technology, and the Internet of Things."

European Parliamentary Research Service, 2016

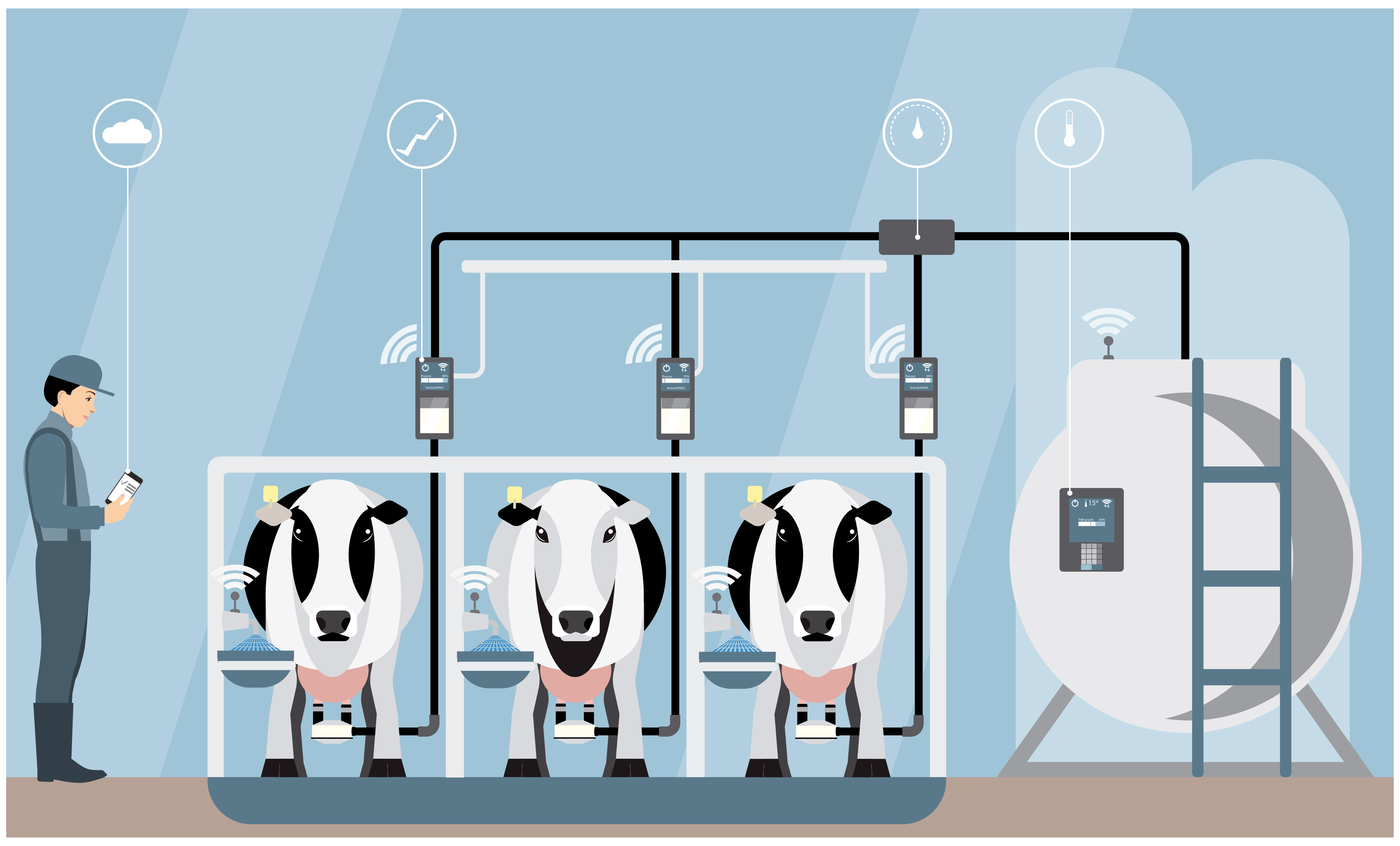

What is Gene Editing?

How CRISPR works

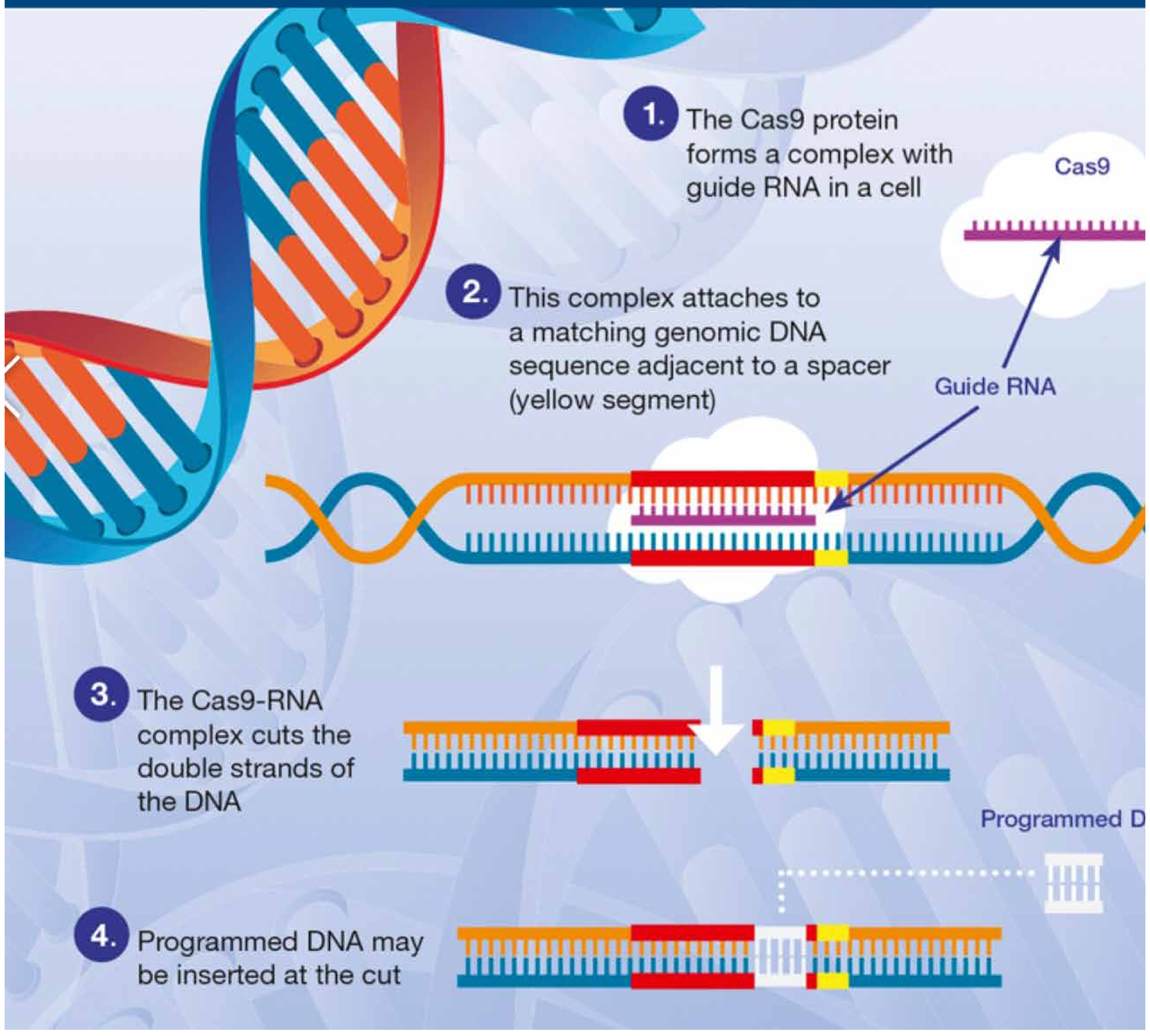

The CRISPR-Cas system "has made editing of the genome much more precise, efficient, flexible and less expensive compared to previous strategies"

National Academy of Sciences, 2017: 1

Gene editing enables scientists to:
add, remove, or alter genetic material at particular locations in the genome.

National Insitute of Health, 2019

Potential applications
"Tomatoes that can sit in the pantry slowly ripening for months without rotting. Plants that can better weather climate change. Cows that no longer grow horns. These organisms might sound far-fetched, but in fact, they already exist, thanks to gene editing."

Doudna and Sternberg (2017:117), A Crack in Creation: The New Power to Control Evolution

Inventors of CRISPR-Cas

The inventors of CRISPR-Cas are from left to right Emmanuelle Charpentier (Max Planck Institute, Berlin) and Jennifer Doudna (University of California, Berkeley). Gene editing was declared scientific breakthrough of the year 2015 by the scientific journal *Science*.

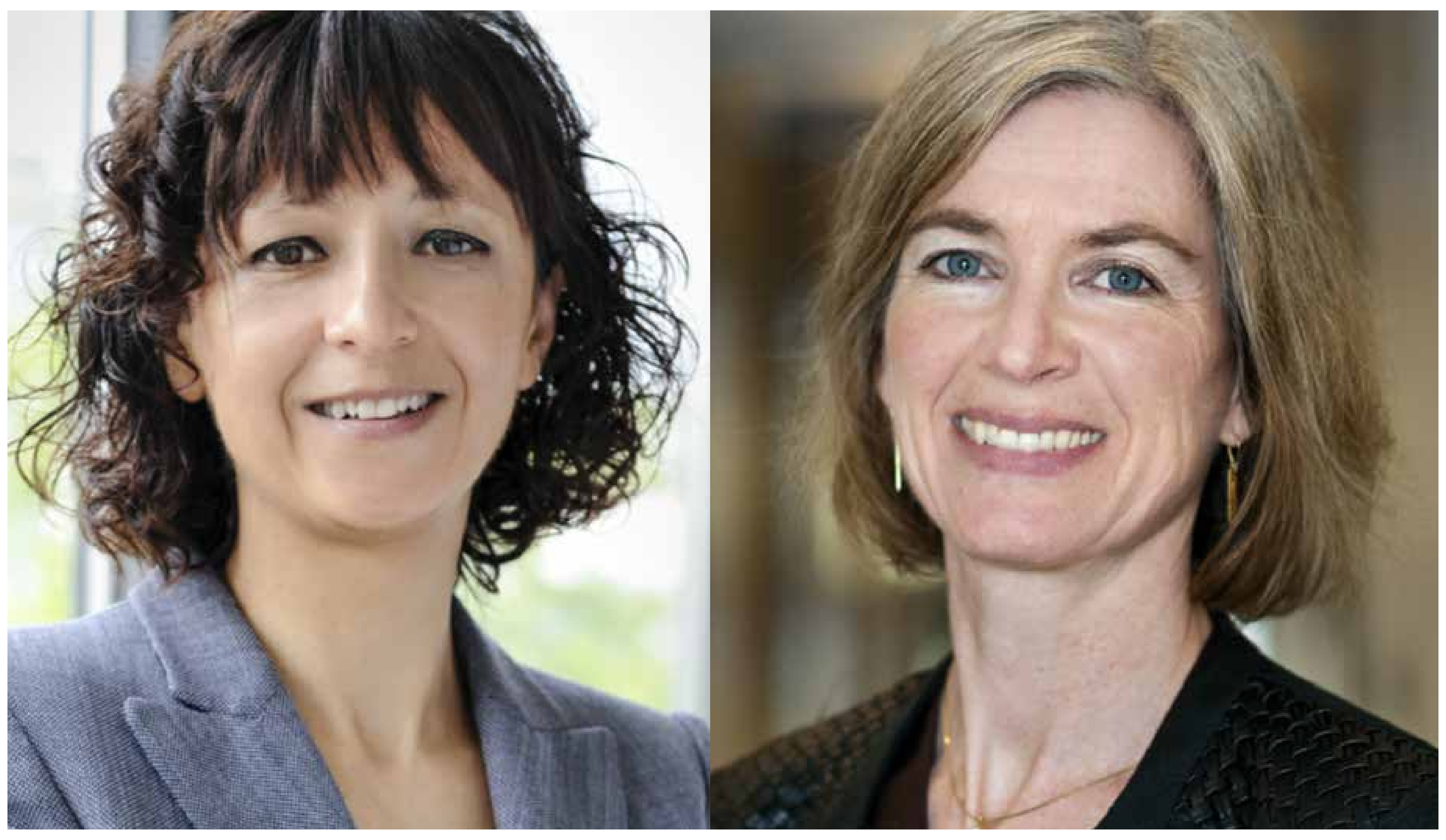

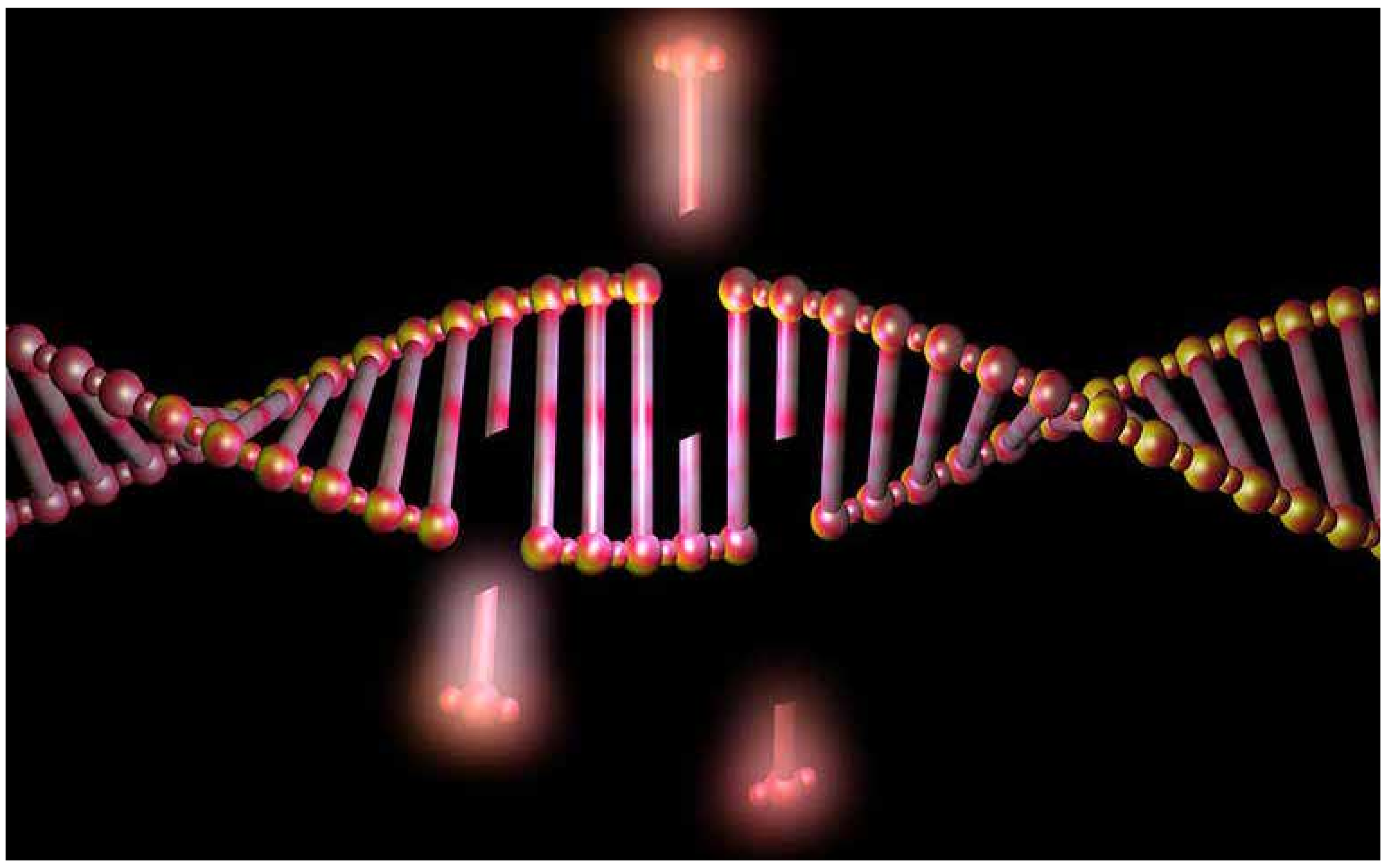

Technical Downsides

Mosaicism

Is incomplete editing, then more than one genotype in one individual can occur.

National Academy of Sciences, 2017

Off-target effects

If Cas9 lacks perfect specificity, "secondary sites may also be cleaved, leading to unwanted mutations."

Carroll, 2019

Agricultural Applications

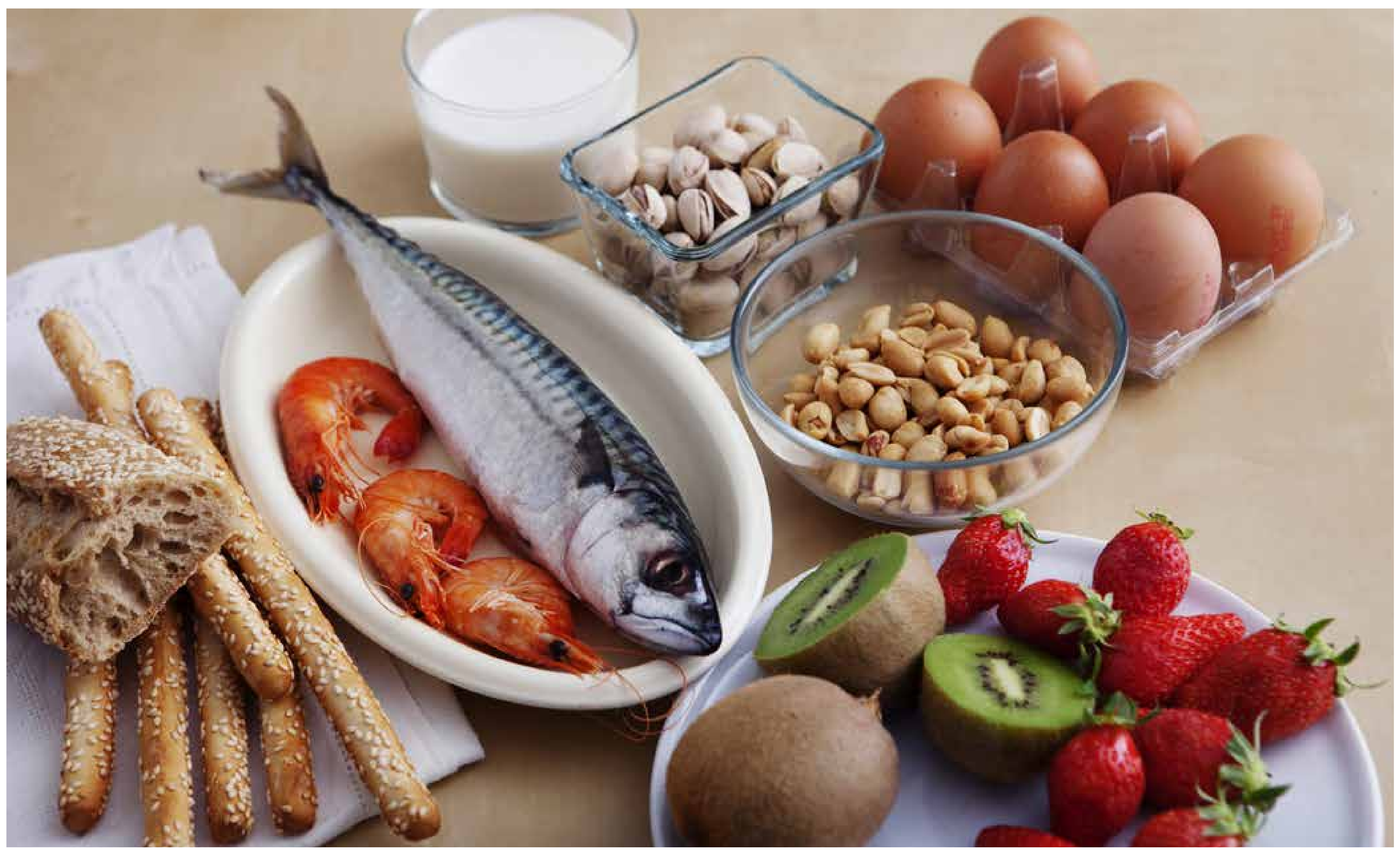

Allergen Free Food

"With CRISPR, it could be possible to make milk, eggs or peanuts that are safe for everyone to eat."

Rodriguez Fernandez, Labiotech, 2019

Disease Resistance

Genus and Beijing Capital Agribusiness cooperate "to research, develop, register and market elite pigs in China that are gene-edited to be resistant to PRRSv, a disease that affects the majority of pigs in China."

PIC website, 2019

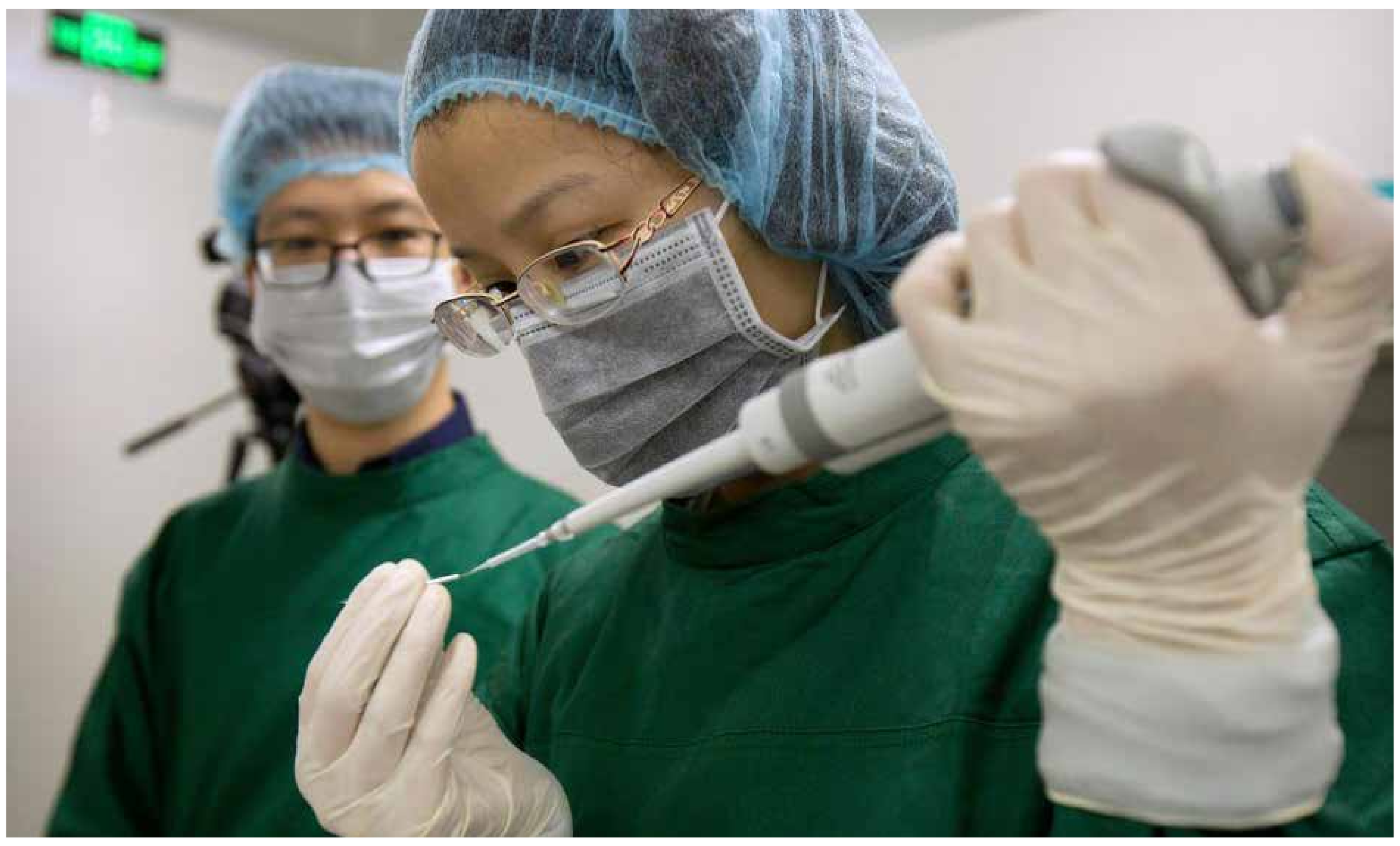

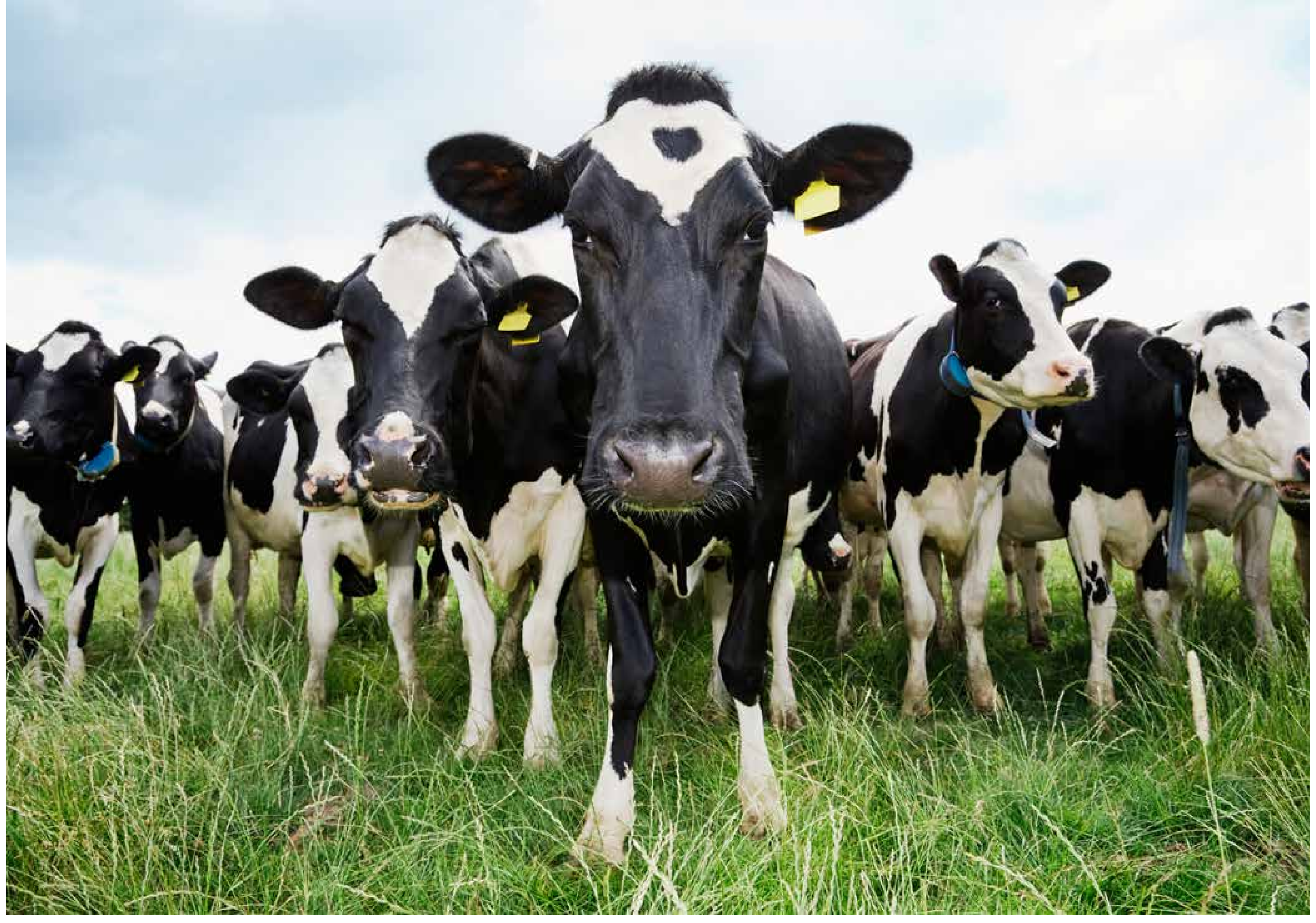

Animal Welfare

FDA scientists tested one of Recombinetics' polled animals and "they discovered its genome contains a stretch of bacterial DNA including a gene conferring antibiotic resistance."

MIT Technology Review, 2019

Recombinetics uses gene editing "to replace the horned gene with a naturally occurring polled gene, providing a direct benefit to the animals' well-being and health." *Recombinetics, 2018*

Climate Change

"Gene editing could offer a novel solution to adapt plant genomes to fight climate stressors and diseases."

Freier, 2019, Medium Biotechnology

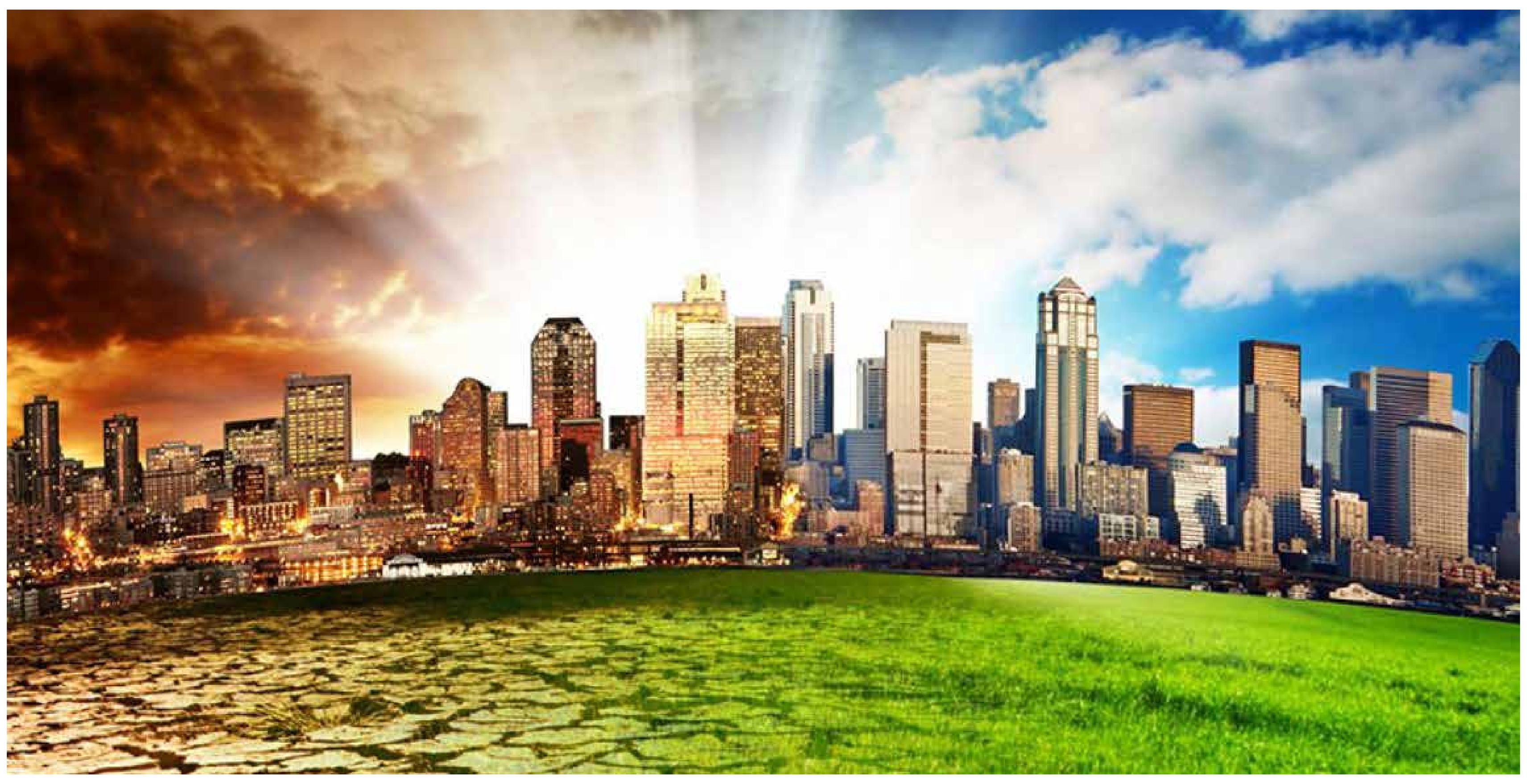

Human Applications

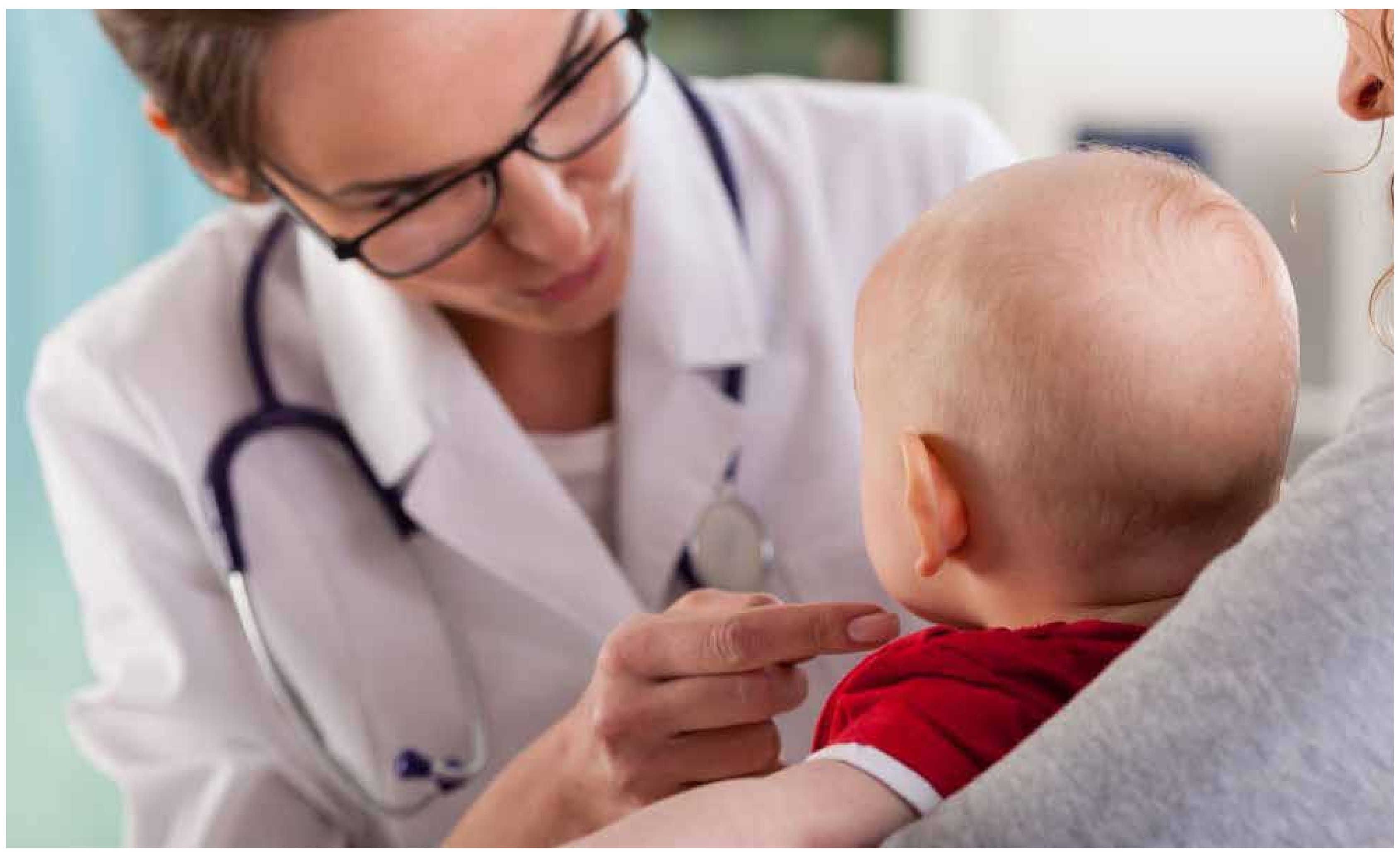

Treatment Applications

Duchenne muscular dystrophy, cystic fibrosis and Huntington's disease, are "all diseases that have known genetic causes, and we now have the technology that can repair those mutations to provide, we hope, patients with a normal life." *Jennifer Doudna*

CNBC, 2016

Research Genes

Gene editing, "is an area of research seeking to modify genes of living organisms to improve our understanding of gene function."

NIH, 2019

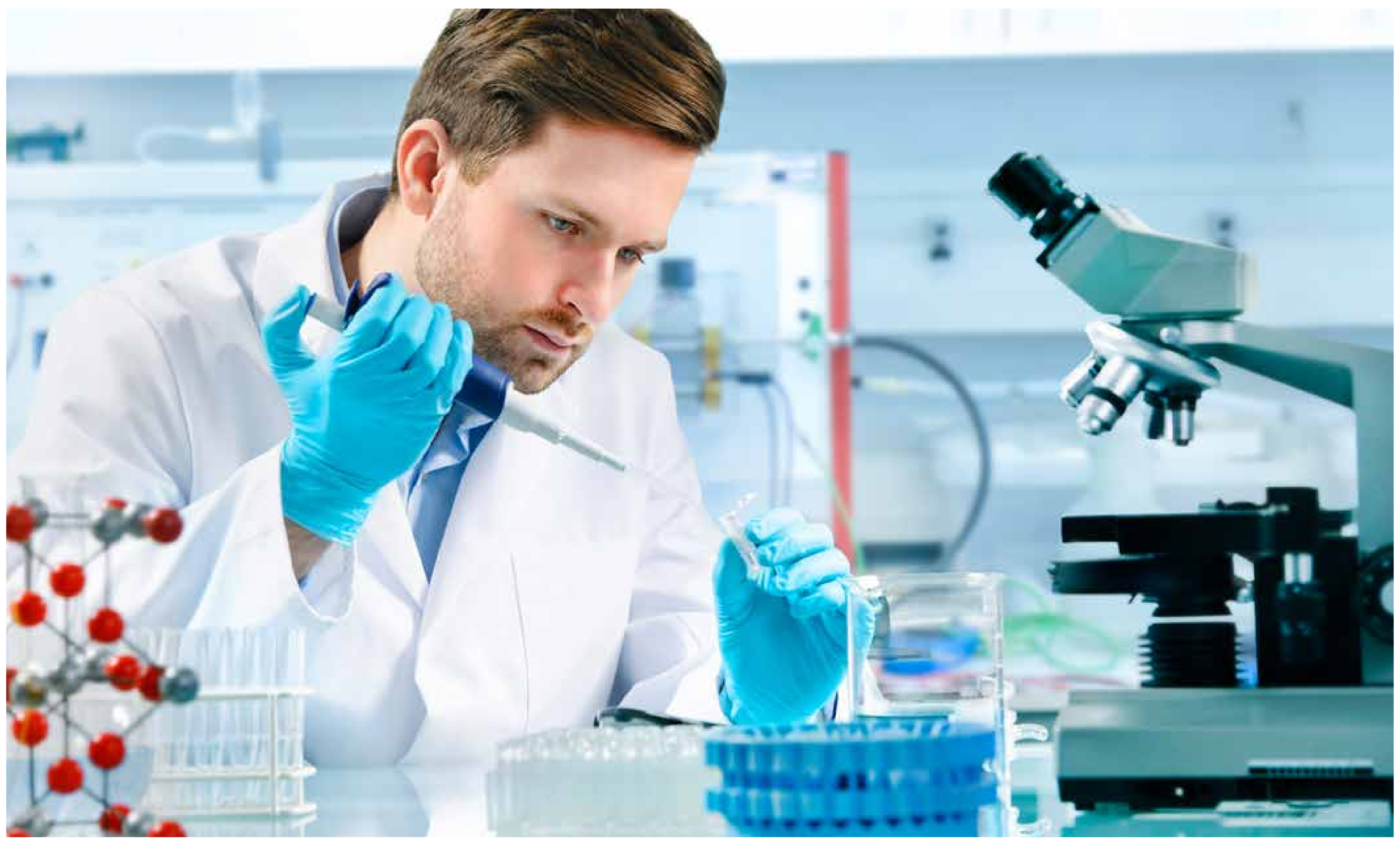

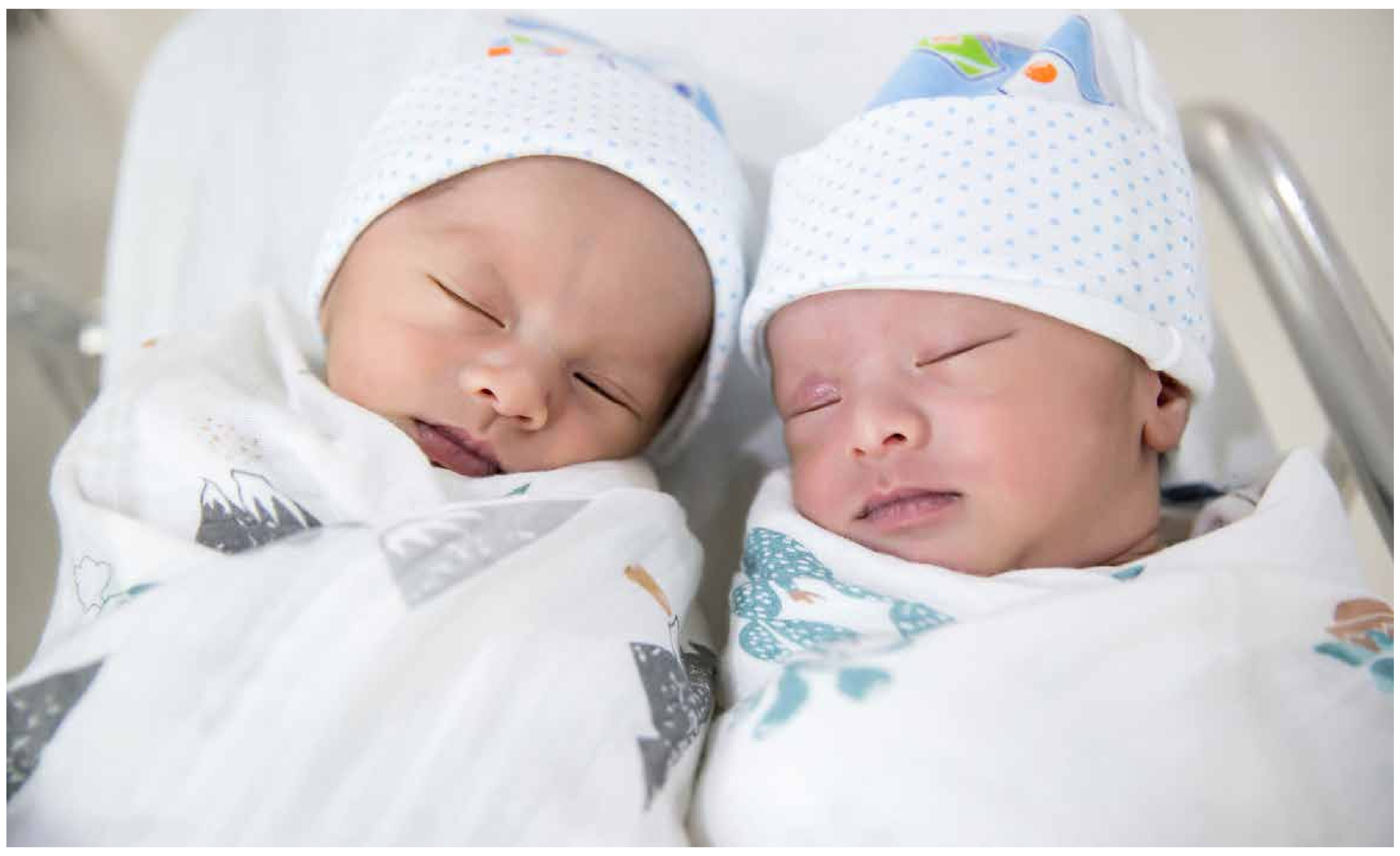

Germline Applications

At the Human Genome Editing Summit in 2018 He Jiankui presented the birth of germline edited Chinese twin girls Lulu and Nana.

"Scientists aren't convinced yet that the HIV protection will be the only thing the CRISPR edit did to the twins' genomes."

Alice Park, Time Magazine, 2018

Gene Editing Humans: It's not just about Curing Diseases

"The temptation to use these technologies to 'enhance' ourselves or our children, or to edit out undesirable traits, will be enormous."

Mildred Z. Solomon (Scientific American) on August 20, 2019

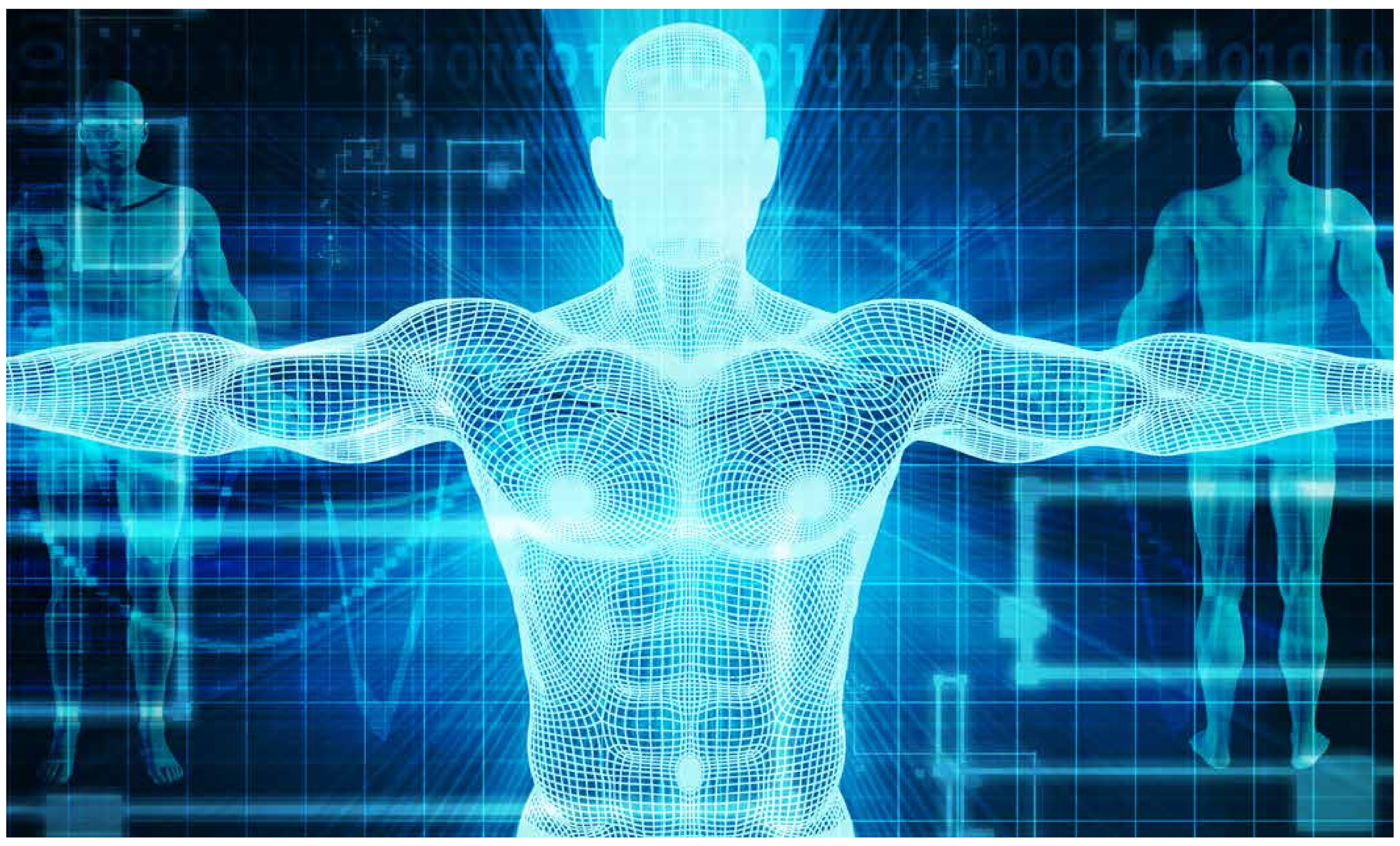

Visions of Gene Editing in Livestock

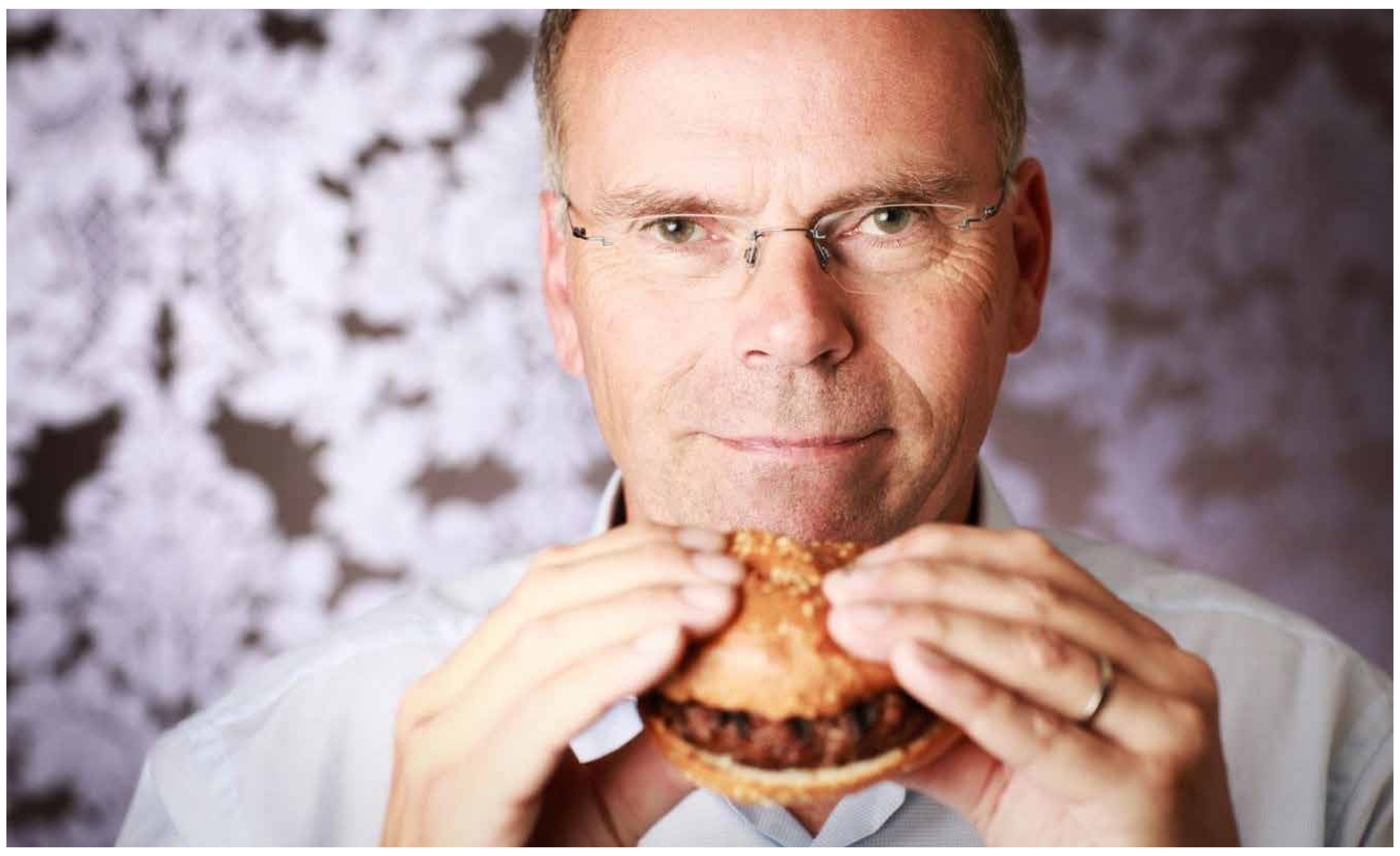

Positive Vision

"Taking advantage of the genome editing revolution, livestock breeding will enter into a new development opportunity and provide humans with higher quality, healthier and lower cost products with richer varieties. Genome editing technology will ignite a revolution in livestock breeding."

Ruan et al. 2017

Negative Vision

"Many applications of these genetic engineering technologies could result in further entrenching the intensive animal farming model, rather than generating true solutions to the serious animal welfare, public health and environmental problems it creates."

Friends of the Earth, 2019

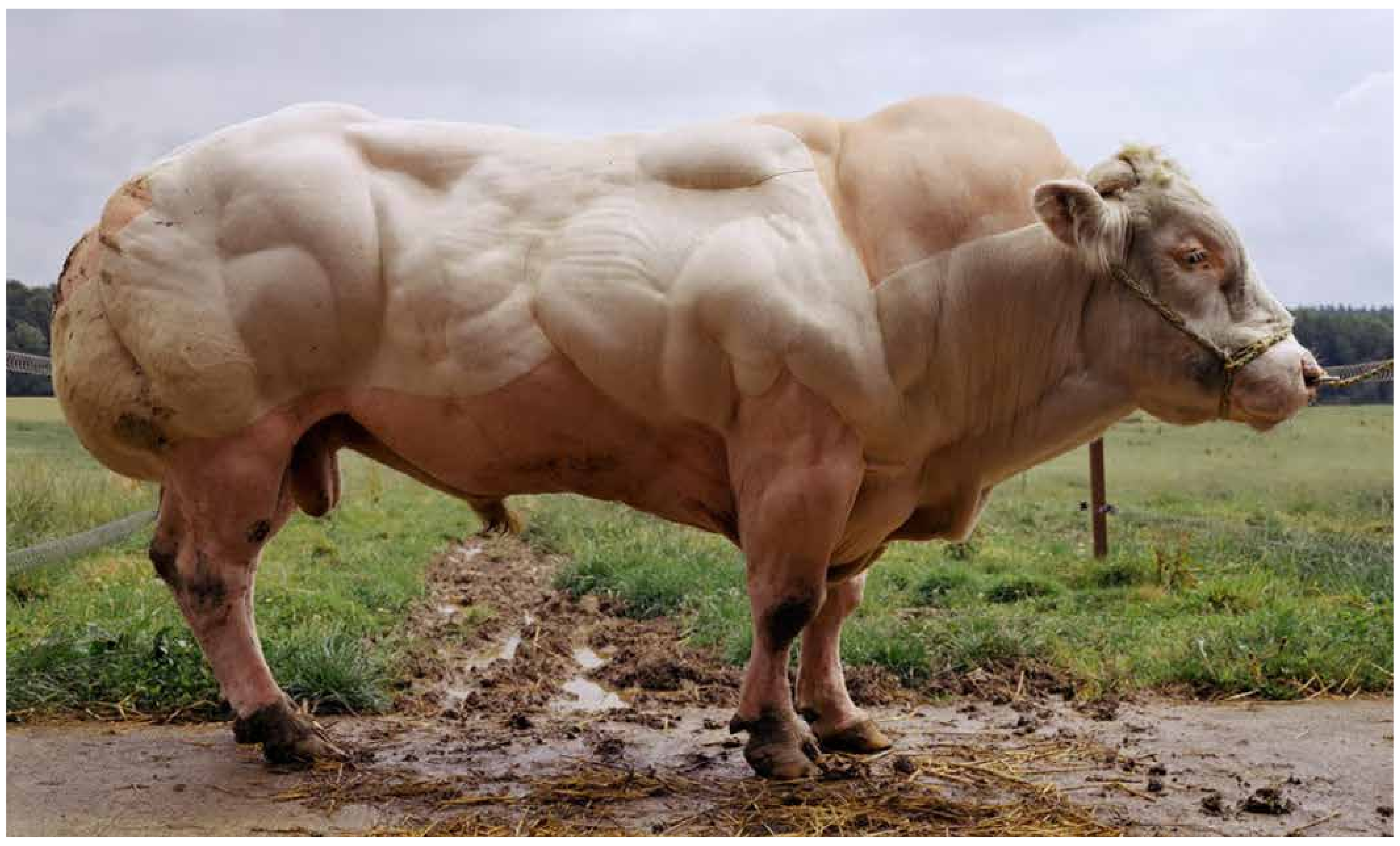

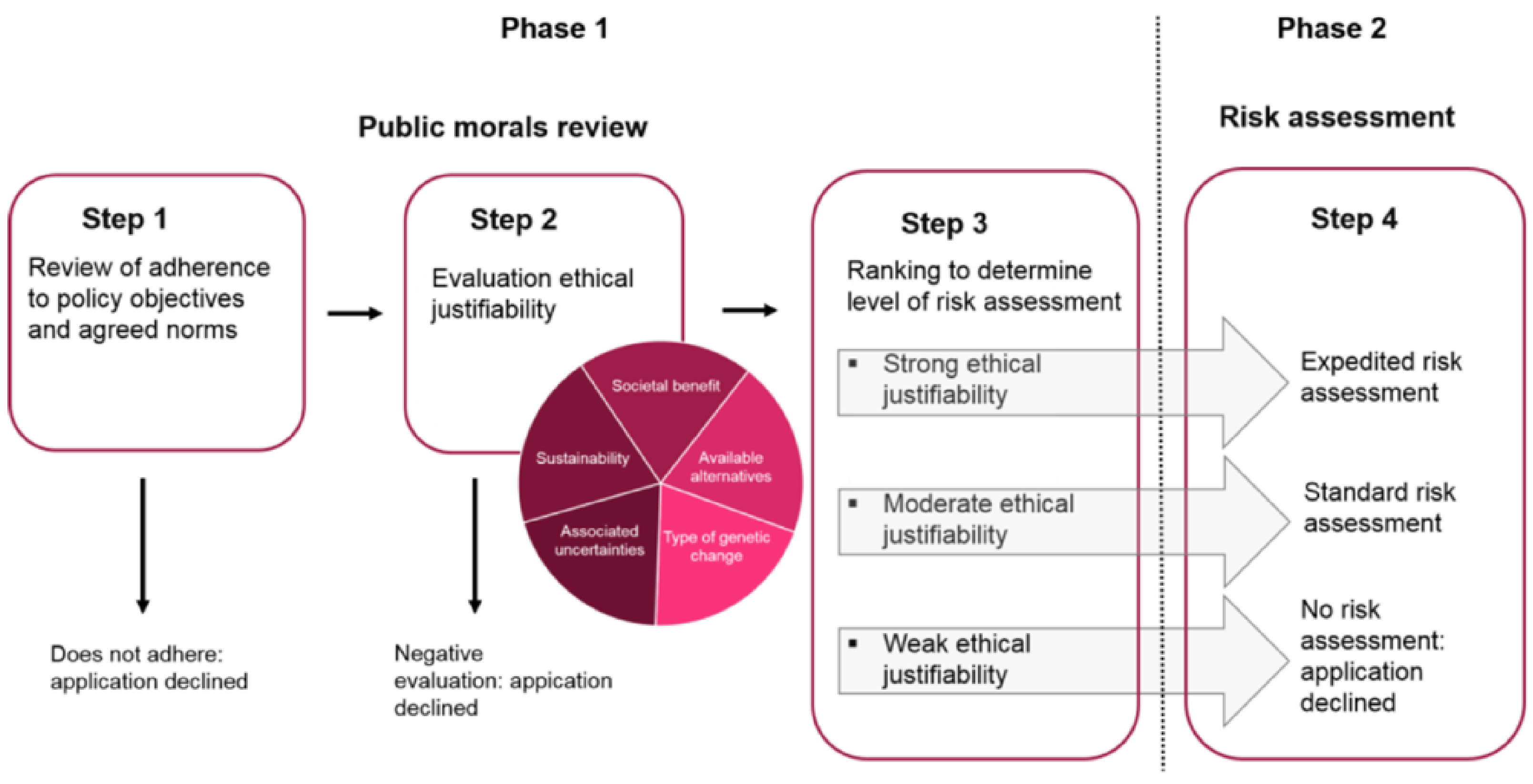

Figure 2 Model 2: level-based model based on broader considerations. These considerations determine the level of ethical justifiability, which in turn determines the level of risk assessment.[97]

Case by Case Vision

"[A] nuanced regulation based on process and product, taking account of risks to public health and the environment, as well as broader societal and ethical questions, is possible."

Rathenau Institute, 2019

Non-ideal Vision

"When compared with a shift towards plant-based diets or to in vitro meat, genetic engineering could be seen as the least attractive choice. ... [But] given the uncertainty surrounding the potential development of in vitro meat and the adoption of plant-based diets, genetic modification remains an important alternative to pursue for the sake of improved animal well-being." *Shriver & McConnachie, 2018*

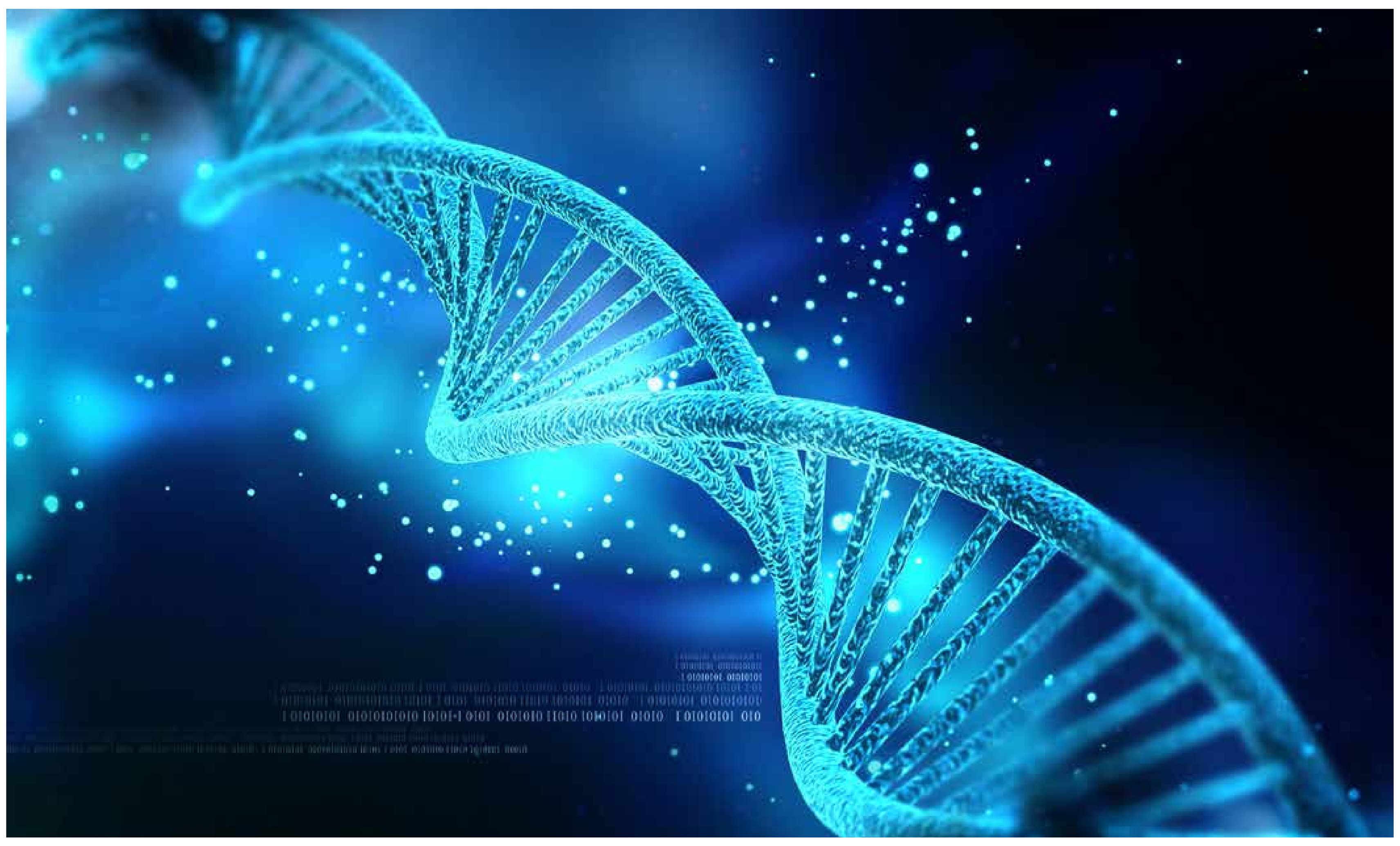

Gene Editing and Governance

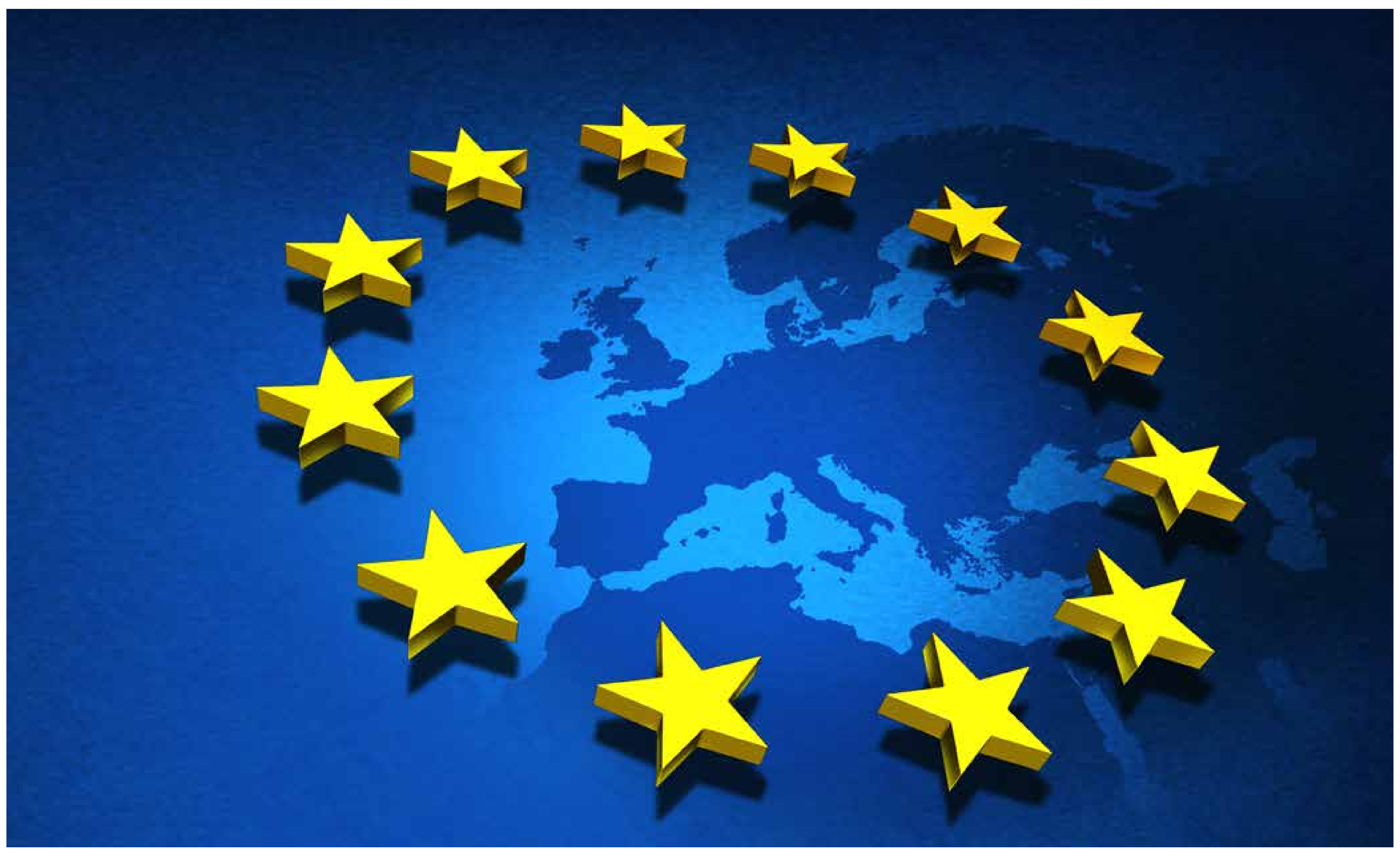

European Union

In the EU, gene editing applications are "subject to an authorization procedure in which they are assessed for potential risks to human health and the environment." (COGEM 2018) This applies "in so far as the techniques and methods ... alter the genetic material of an organism in a way that does not occur naturally."

EU Court of Justice, 2018

United States

"In the US, the assessment of the risk posed by the resulting organisms to human beings, animals or the environment is predominantly based on the end product and not the technological process."

Sprink et. al., 2016

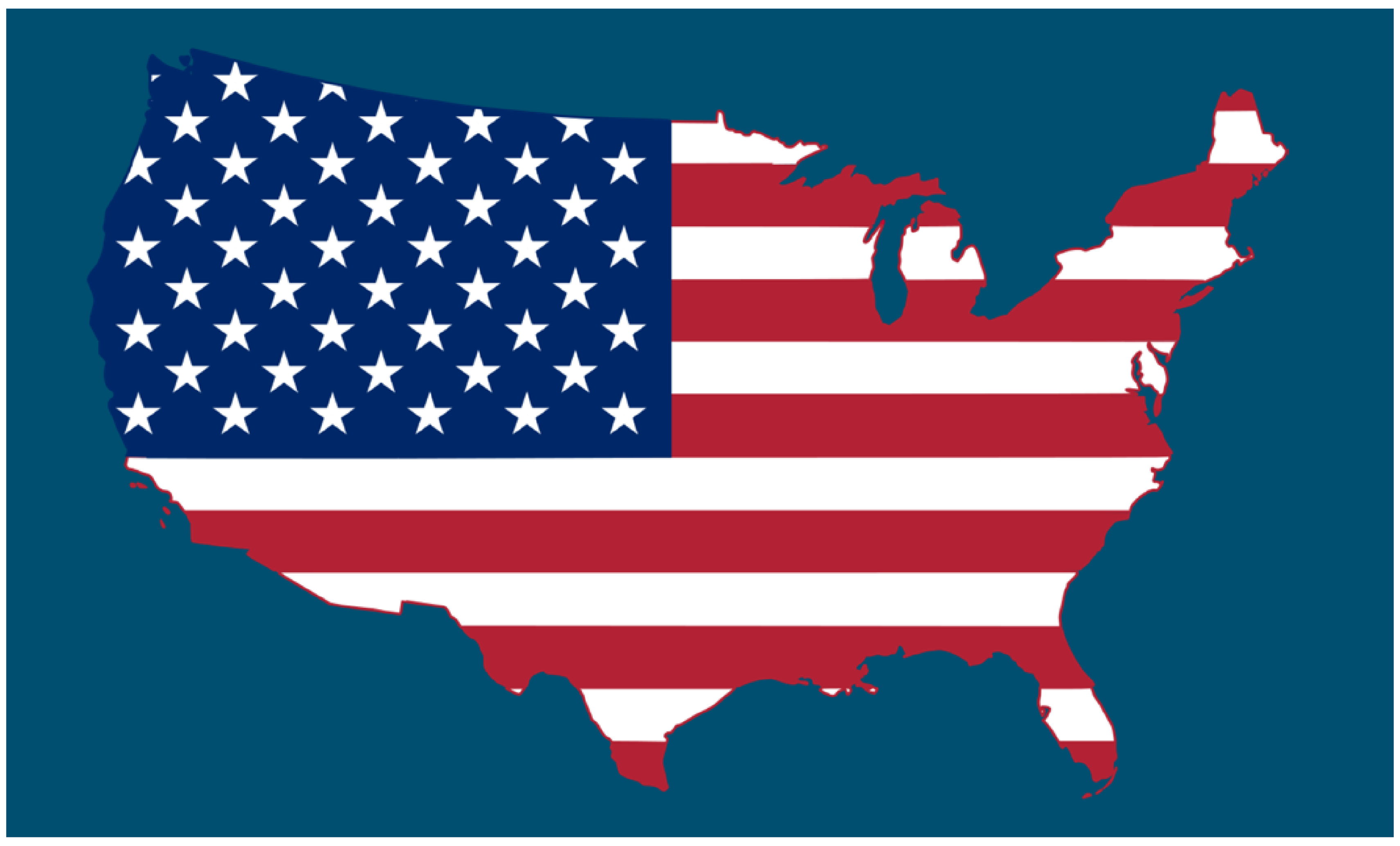

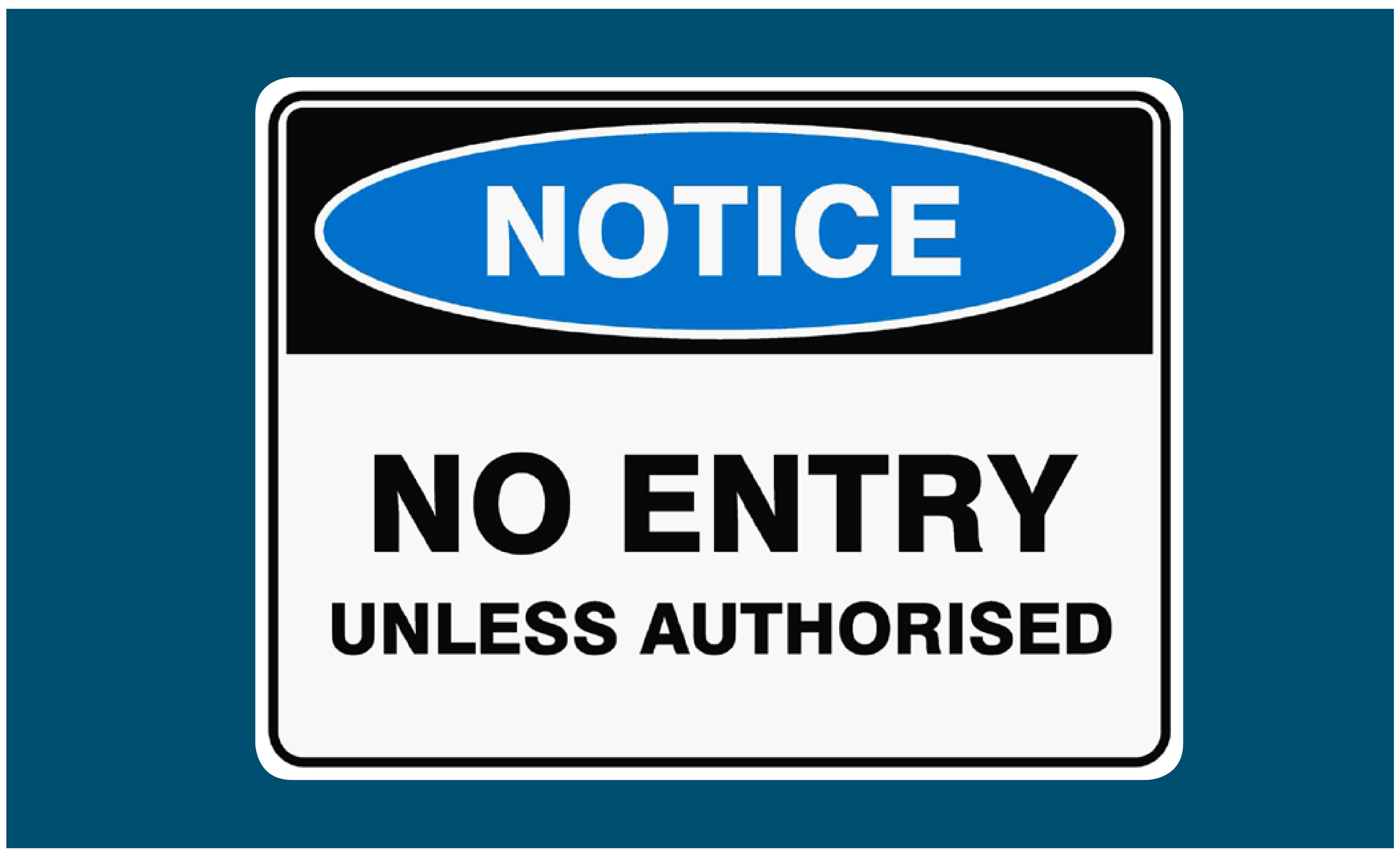

'No unless' Policy

"Licences for animal biotechnology applications are granted if the activities have no unacceptable consequences for the health or welfare of animals and there are no ethical objections to the activities. There must also be no realistic alternatives available for the proposed research or the proposed practical applications."

COGEM, 2018

Public Engagement

"Actively involving citizens and acknowledging their concerns and interests are essential for a responsible integration of innovative technologies in society. Factors such as the goal of a specific innovation, the contribution to challenges to society, and the desirability of GMOs as a solution to these challenges need to be given sufficient attention in the discussion." *Rathenau Institute, 2019*

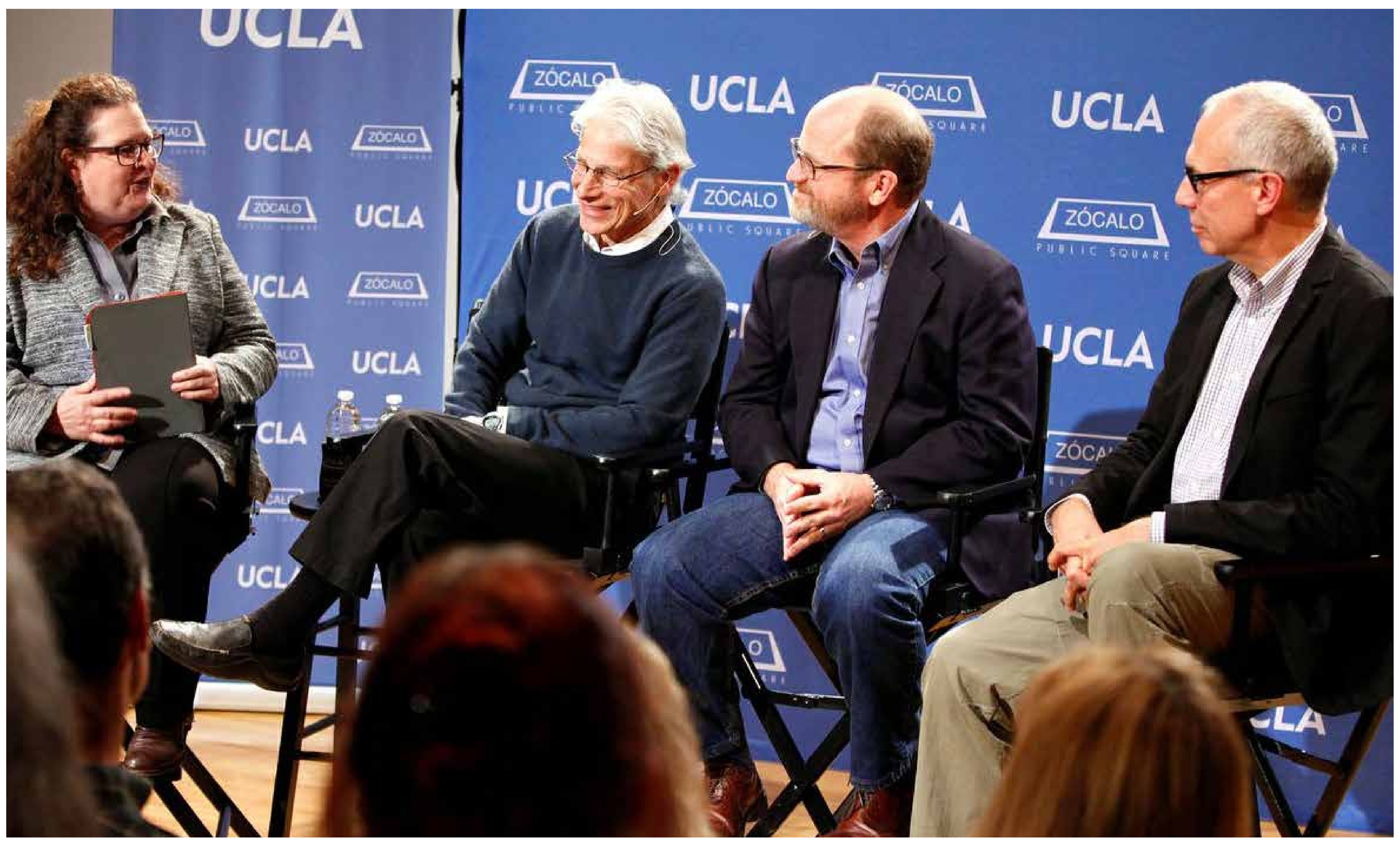

Supplement: sj-pdf-1-pus-10.1177_09636625221111900 – Supplemental material for Imagined futures for livestock gene editing: Public engagement in the Netherlands [file sj-pdf-1-pus-10.1177_09636625221111900.pdf]
